# Tumor-derived PCSK9-enriched exosomes reprogram adipocytes to drive metabolic dysregulation and immune evasion in triple-negative breast cancer

**DOI:** 10.1038/s41419-026-09027-y

**Published:** 2026-07-03

**Authors:** Duanyang Zhai, Yawei Shi, Yuanjian Fan, Shaoquan Zheng, Mengmeng Zhang, Shuling Zhou, Nan Shao, Yunjian Zhang, Jihong Cui, Ying Lin

**Affiliations:** 1 Breast Disease Center, The First Affiliated Hospital of Sun Yat-sen University, Guangzhou, Guangdong China; 2 Institute of Precision Medicine, The First Affiliated Hospital of Sun Yat-sen University, Guangzhou, Guangdong China

**Keywords:** Biogeochemistry, Cancer

## Abstract

Triple-negative breast cancer (TNBC) is characterized by aggressive behaviors, limited treatment options, and poor prognosis. Adipocytes, the predominant cellular component in the tumor microenvironment (TME) of breast cancer, interact bidirectionally with tumor cells, influencing tumor progression and immune evasion. Understanding the interactions between TNBC cells and adipocytes within TME is crucial for identifying new therapeutic targets. We employed murine models and co-culture systems to investigate the effects of adipocyte-TNBC cell interactions on lipid metabolism, signaling pathways, and immune evasion mechanisms. Our findings revealed that TNBC cells utilized PCSK9-enriched exosome transfer to induce the formation of cancer-associated adipocytes (CAAs) from adipocytes. These CAAs, characterized by altered lipid content and a pro-inflammatory secretory profile, contributed to a tumor-promoting environment. Additionally, CAAs enhanced the immune evasion properties of TNBC by modifying the metabolic pathways of tumor cells. In depth, CAAs induced fatty acid oxidation (FAO) in TNBC cells, which facilitated G3BP1-induced stabilization of PCSK9 and promoted OPTN-mediated autophagic degradation of the co-stimulatory molecules CD80 and CD86, which are essential for T cell activation. Our study identifies PCSK9 as a central mediator in the bidirectional interactions between TNBC cells and adipocytes, influencing tumor progression and immune evasion. These insights suggest that targeting the PCSK9 pathway could provide new therapeutic opportunities for TNBC, potentially transforming treatment approaches and improving patient outcomes. Further investigation into the mechanisms by which PCSK9 regulates these processes may yield novel combination strategies to enhance the efficacy of immunotherapy in TNBC.

**Figure:**
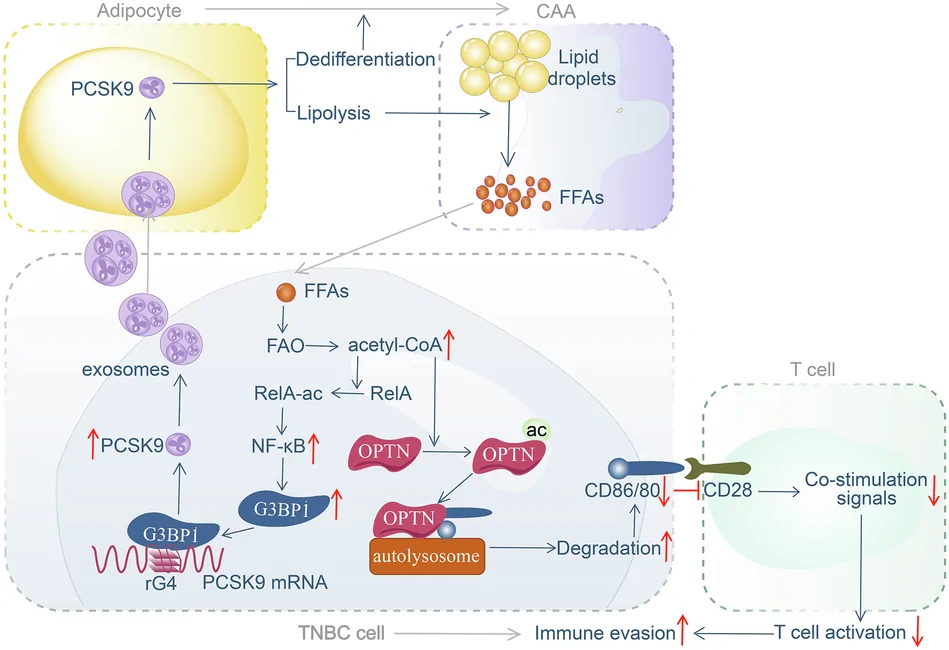

## Introduction

Triple-negative breast cancer (TNBC), representing 15–20% of all breast cancers, is a biologically and clinically heterogeneous malignancy defined by the lack of estrogen receptor (ER), progesterone receptor (PR), and human epidermal growth factor receptor 2 (HER2)[1]. TNBC patients experience the poorest prognosis due to the higher incidence of distant metastasis and recurrence compared to other breast cancer subtypes. Moreover, TNBC continues to pose significant challenges in treatment options due to the lack of canonical therapeutic targets [2, 3]. Recently, immune-checkpoint inhibitors (ICIs) have emerged as a promising therapy for TNBC; however, only a small subset of TNBC patients respond to ICIs [4–6]. The reasons for the limited response to ICIs are multifactorial. Immune evasion mechanisms significantly contribute to the ineffectiveness of immunotherapy in treating cancers. A crucial factor is the immunosuppressive tumor microenvironment (TME), which hinders effective immune responses [7].

TME is a multifaceted, heterogeneous system comprising diverse cell types and extracellular components, including immune cells, endothelial cells, cancer-associated fibroblasts, and adipocytes. The intricate interplay among these components operates in a highly coordinated manner, shaping the TME’s role in tumor development and treatment resistance [8–14]. Adipocytes are the most abundant cellular component of the TME in breast cancer, especially in obese tumor patients, and interact bidirectionally with tumor cells [15]. Peritumoral adipocytes, exhibiting altered metabolic and functional phenotypes characterized by reduced lipid content and loss of adipocyte original identities, are known as cancer-associated adipocytes (CAAs) [16, 17]. CAAs act as active endocrine cells, secreting adipokines and proinflammatory cytokines such as interleukin 6 (IL-6), interleukin 1β (IL-1β), leptin, tumor necrosis factor-α (TNF-α), leptin, and adiponectin to impact breast cancer cell behavior and promote tumor progression [18–20]. Additionally, a nutritional exchange between CAAs and tumor cells modifies their metabolic pathways, such as glucose and lipid metabolism, thereby supporting the extreme energy and biosynthetic demands of tumor cells [21–24]. Furthermore, adipocytes can influence the immune landscape of the TME. They affect immune cell function and recruitment, potentially leading to an immunosuppressive environment that helps tumors evade immune surveillance [17, 25–27]. Additionally, obesity, a highly significant risk factor for breast cancer progression [28, 29], reshapes the metabolic landscape of the TME, blunting anti-tumor immune responses and promoting tumor growth [30, 31]. Despite these insights into the role of adipocytes in tumor progression and immune evasion, the key molecular mechanism driving the crosstalk between adipocytes and tumor cells, and how this reciprocal interaction helps safeguard tumor cells from immune surveillance to promote their progression, remains poorly understood.

In this article, we found that PCSK9 is a key player in the bidirectional interactions between adipocytes and breast cancer cells, affecting tumor cell immune evasion and tumor progression. TNBC cells transfer PCSK9-containing extracellular vesicles (EVs) to adipocytes, crucial for transforming adipocytes into CAAs. Reciprocally, CAAs trigger lipid metabolic reprogramming in TNBC cells, leading to enhanced fatty acid oxidation (FAO), which, in turn, elevates PCSK9 expression via the NF-κB pathway in TNBC cells. This FAO enhancement in TNBC cells, facilitated by co-culture with adipocytes, dampens T cell activation by inducing autophagic degradation of CD80/CD86 on tumor cells, thereby preventing the costimulatory signals required for T cell activation and driving immune evasion in TNBC. Mechanistically, OPTN, as the primary cargo receptor mediating CD80/CD86 autophagic degradation, its acetylation and affinity to CD80/CD86 were increased with the elevated FAO level in TNBC cells under adipocyte co-culture. Our findings further highlight PCSK9 as a potential immunotherapeutic target for breast cancer treatment.

## Methodology

### Animal experiment

All animal procedures were conducted in strict accordance with institutional animal welfare guidelines and approved by the Institutional Animal Care and Use Committee (IACUC) of the First Hospital of Sun Yat-sen University. Eight female BALB/c mice (aged at 6–8 weeks) were procured from Sun Yat-sen University and housed in a controlled environment at 22–28 °C with 60% humidity, with ad libitum access to food and water. Following a one-week acclimatization period, mice were randomly assigned to two groups (4 mice each group): a standard diet group (12% calorie fat content) and a high-fat diet group (45% calorie fat content). Diets were maintained for 12 weeks, during which time changes in blood sugar levels 1 and 2 hours after meals were measured by a glucose meter every 30 min. Serum fatty acid oxidation (FAO) levels were determined using a mouse-specific FAO ELISA kit. Body fat composition was analyzed using a QMR awake body composition analyzer. Subsequently, 1 million TNBC cells (4T1) were injected via the tail vein using a Matrigel (1:1) mixture. Or a mixture of 1 × 10^6^ cells was inoculated in the second pair of mammary fat pads on the right side of mice. Tumor growth was monitored starting from the 7th day post-injection, and tumor and lung tissues were collected for further analysis. The operator and evaluator responsible for tumor detection and Hematoxylin and Eosin analyses of lung metastases were blinded to the experimental conditions.

### Cell culture and drug treatment

Both adipocytes (3T3-L1) and TNBC cells (MDA-MB-231 and 、4T1) were purchased from ATCC. All cell lines were authenticated via STR profiling and were tested for mycoplasma contamination (negative) before use. Cells were cultured in RPMI 1640 medium supplemented with 10% fetal bovine serum and 1% penicillin/streptomycin at 37 °C with 5% CO_2_. For cell suspension preparation, cells were digested, centrifuged to remove medium, washed with PBS, resuspended in fresh medium, and adjusted to a density of 5 × 10^6^ cells/mL. Adipocytes were co-cultured with breast cancer cells, with adipocytes (500 μL) in the upper layer and breast cancer cells (1500 μL) in the lower layer for at least 24 h. Following co-culture, adipocytes co-cultured with TNBC cells were treated with a 200 μM FAO inhibitor (Etomoxir, Catalog #E1905, Sigma-Aldrich) for 0.5 hours, and cells were collected for subsequent analysis. In addition, the tumor cells were treated with BafA1 (200 nM), CMX (100 μg/mL), and MG132 (10 μM), respectively, to detect autophagy and protein degradation.

### T cell isolation and activation

Using Ficoll gradient centrifugation, human peripheral blood mononuclear cells (PBMCs) from healthy donors and spleen lymphocytes (SLs) from C57BL/6 mice (purchased from NCI) were isolated. For T cell activation, PBMCs and SLs were stimulated by RPMI-1640 medium (Gibco) containing anti-CD3 (100 ng/ml, Biolegend; Cat No.317326), anti-CD28 (100 ng/ml, Biolegend; Cat No.302914), and recombinant IL-2 (10 ng/ml, Peprotech, 200-02) for 5–7 d.

### CFSE proliferation assay

The impact of TNBC cells with or without adipocyte coculture on T cell proliferation was examined by CFSE (Invitrogen; Cat No. C34554). In brief, CFSE-labeled T cells derived from PBMCs or SLs were cocultured with adipocyte-educated or parental MDA-MB-231 cells for 4 days. After that, CFSE density in the harvested T cells was assessed via flow cytometry.

### Cell transfection

TNBC cells were inoculated into a six-well plate and transfected with Lipofectamine 3000 (L3000075, Invitrogen, Carlsbad, CA, USA) for PCSK9 interference vectors, and controls were performed according to the manufacturer’s protocol. 48 h after transfection, cells were collected for follow-up experiments.

### Bodipy lipid droplet staining

Bodipy dye (ThermoFisher, D3922) was employed to stain breast cancer cells pre- and post-co-culture, adhering strictly to the manufacturer’s guidelines. The lipid droplet content was microscopically examined, and the percentage of cells exhibiting lipid droplet uptake before and after co-culture was assessed via flow cytometry.

### Oil red staining

Oil Red staining was conducted to assess lipid droplet levels in breast cancer cells before and after co-culture. Briefly, cells were fixed with a formaldehyde-calcium solution, then frozen-sectioned and dried. After a brief rinse with 50% ethanol, Oil Red O staining solution was prepared by dissolving 0.5 g of Oil Red O in 100 mL of 50% ethanol. Sections were immersed in the staining solution for 8 min, then differentiated in 50% ethanol and terminated in tap water. Counterstaining with hematoxylin was performed for nuclear visualization, and sections were mounted in glycerol-gelatin for lipid droplet observation under a microscope.

### Triglyceride content analysis

Triglyceride content in adipocytes was quantified using the triglyceride Assay Kit (ab65336). Sample and standard wells were prepared according to the kit instructions. Experimental buffer and lipase were added, followed by a 20-minute incubation period. Subsequently, the triglyceride reaction mixture was added and incubated for 60 minutes. Finally, triglyceride levels were measured using a microplate reader.

### Reverse transcription quantitative polymerase chain reaction (RT-qPCR) analysis

Total RNA was extracted from adipocyte and triple-negative breast cancer cell samples using TRIzol Reagent (Invitrogen, Carlsbad, CA, USA). Subsequently, cDNA synthesis was performed using the PrimeScript Reverse Transcription Kit (Takara, Shiga, Japan), utilizing 1 μg of total RNA. SYBR Premix Ex Taq II (Takara) was used for qPCR. Relative gene expression was calculated using the comparative cycle threshold (ΔΔCt) method, with GAPDH as the reference gene.

### Western blot

Adipocytes and breast cancer cells were lysed in 1% Nonidet P-40 lysis buffer supplemented with a protease inhibitor mixture (Sigma-Aldrich) and 1 mM sodium orthovanadate (Sigma-Aldrich). Total protein lysates (20 μg) were resolved by SDS-PAGE, transferred onto nitrocellulose membranes, and probed with specific antibodies overnight at 4 °C. The following primary antibodies were used: MMP11 (1/500, ab236510, Abcam), Calnexin (1/1000, ab22595, Abcam), HSP70 (1/500, ab2787, Abcam), CD63 (1/1000, ab134045, Abcam), PCSK9 (1/2000, ab181142, Abcam), G3BP1 (1/5000, ab181149, Abcam), CD86 (1/1000, ab239075, Abcam), CD80 (1/1000, ab134120, Abcam), IFN-γ (1/1000, sc-373727, Santa Cruz Biotechnology), Granzyme B (1/1000, ab255598, Abcam), anti-RelA-ac (1/1000, ab19870, Abcam), anti-RelA (1/1000, ab32536, Abcam), β-actin (1/1000, ab8226, Abcam), and IgG (1/10000, ab205718, Abcam). After washing, membranes were incubated with appropriate secondary antibodies and visualized using an enhanced chemiluminescence substrate (Pierce).

### ELISA assays

Resistin measurement: Supernatants from adipocytes were collected, and Resistin levels were quantified using a Mouse Resistin ELISA kit (ml001903, Mlbio) following the manufacturer’s protocol. The assay included sample addition, incubation, reagent preparation, washing, enzyme addition, subsequent incubation, washing, color development, and termination. Absorbance was measured at 450 nm using an ELISA reader.

APN measurement: APN levels were determined using a Mouse Adiponectin (APN) ELISA kit (CB14814-Mu, COIBO). The microplate was coated with the target antibody to create a solid-phase carrier. Standard samples or specimens were added to the wells, followed by incubation, washing, and the addition of substrate A and B. After light-protected incubation, the reaction was terminated, and absorbance was measured at 450 nm using an ELISA reader.

IL-6 measurement: Mouse Interleukin 6 (IL-6) levels were determined using a Mouse IL-6 ELISA kit (ml098430, Mlbio) according to the manufacturer’s instructions. Absorbance was measured at 450 nm using an ELISA reader.

IL-1β measurement: Mouse Interleukin 1β (IL-1β) levels were determined using a Mouse IL-1β ELISA kit (ml098416, Mlbio) following the manufacturer’s instructions. The assay involved sample addition, incubation, washing, subsequent addition of substrate A and B, light-protected incubation, and termination. Absorbance was measured at 450 nm using an ELISA reader.

ATP measurement: ATP levels were determined using an ATP content test kit (ml092826, Mlbio) following the manufacturer’s instructions. Absorbance was measured at 636 nm using an ELISA reader.

Acetyl-CoA measurement: Acetyl-CoA levels were determined using an Acetyl-CoA test kit (ml077327, Mlbio) according to the manufacturer’s instructions. Absorbance was measured at 340 nm using an ELISA reader.

FAO measurement: FAO levels were determined using a FAOBlue (Fatty Acid Oxidation Detection Reagent) (FDV-0033, Funakoshi) assay according to the manufacturer’s instructions. Absorbance was measured at 405 nm.

Blood glucose measurement: Blood glucose levels were determined using a Mouse Glucose kit (TW10647, TW-reagent) following the manufacturer’s instructions. Absorbance was measured at 450 nm using an ELISA reader.

### Extracellular vesicle extraction and identification

Extracellular vesicles were isolated from adipocytes using an ultracentrifugation-based method. Briefly, 50 mL of cell culture supernatant was initially centrifuged at 500 × *g* for 10 min (Beckman Allegra X-15R, rotor SX4750, k = 1200, Beckman Coulter) at 4 °C. The resulting supernatant was carefully transferred to a new centrifuge tube and centrifuged again at 2000 × *g* for 15 min (rotor SX4750, k = 1100) at 4 °C. Subsequently, the supernatant was transferred to a fresh tube and centrifuged at 10,000 × *g* for 30 min (Beckman Optima XE-100, rotor 70 Ti, k = 150) at 4 °C. The supernatant obtained was then filtered through a 0.22 μm membrane to remove remaining debris. The filtered supernatant was collected and transferred to a clean ultracentrifuge tube, then centrifuged at 100,000 × *g* for 70 min (SW32 Ti swinging-bucket rotor, k = 133) at 4 °C. After centrifugation, the supernatant was discarded, and the resulting pellet containing extracellular vesicles was carefully resuspended in 5 mL of phosphate-buffered saline (PBS). This suspension underwent further centrifugation for purification, repeated once more for thorough washing. Finally, the purified pellet was resuspended in 50 μL of PBS and transferred to a 1.5 mL centrifuge tube for long-term storage at –80 °C.

### Transmission electron microscopy (TEM)

Extracellular vesicles were fixed with glutaraldehyde (Sigma-Aldrich, G6257), and 10 μL of the vesicle suspension was deposited onto a Ted Pella copper grid. After adsorption for 10 minutes at room temperature, excess liquid was blotted with filter paper. Subsequently, 10 μL of 2% phosphotungstic acid solution (pH = 6.5) was applied to the grid, and the vesicles were stained for 2 minutes at room temperature. Excess staining solution was removed with filter paper, and the copper grid was air-dried before observation under a TEM.

### Nanoparticle tracking analysis

Extracellular vesicles were diluted in PBS to a concentration of 1 × 10^6^ vesicles per milliliter. The diluted vesicle solution was introduced into a nanoparticle tracking analysis instrument using a syringe. A laser beam was directed through the sample chamber, and vesicles were visualized using a microscope equipped with a camera. Brownian motion of the vesicles was recorded, and their hydrodynamic diameters were calculated using the Einstein equation.

### Extracellular vesicle uptake experiment

Extracellular vesicles (EVs) containing 200 μg of protein in 150 μL PBS were used as the baseline. A 5 μL aliquot of EvLINK probe specific for EV staining was added and incubated at room temperature, shielded from light, with gentle agitation for 30 min. The sample was subsequently purified using chromatography (column conditions: CORE400 packing material, PBS buffer at pH 7.4 as the mobile phase; centrifugation conditions: 20–25 °C, 1000–2000 × *g* for 1–3 min, followed by collection of the supernatant) to remove residual dye, resulting in purified EVs.

Cells were seeded at a density of 2–5 × 10^4^ cells per well in a 24-well plate and cultured for 24 h. Labeled EVs were added to the wells at a final protein concentration of 50 μg/mL and further incubated for 24 h. After removing the culture medium, cells were washed 2–3 times with DPBS. A 5 μL aliquot of the specific probe for cell staining diluted in 500 μL DPBS was added to each well and incubated at room temperature, shielded from light, for 30 min. Cells were then washed again with DPBS to remove excess dye. Finally, cells were fixed with 4% paraformaldehyde (PFA) for 30 min and subjected to nuclear staining followed by confocal imaging.

### PathCards analysis

Proteins related to lipid digestion and mobilization were identified using the PathCards database (https://pathcards.genecards.org/).

### Mass spectrometry analysis

Extracellular vesicles from normal breast epithelial cells and triple-negative breast cancer cells underwent LC/MS analysis. In short, the exosome-like vesicles were resuspended in 100 μL of PBS, 2 μL Triton X-100, and 5 μL phenylmethylsulfonyl fluoride with vortexing to dissolve the vesicles. The insoluble fraction was pelleted by centrifugation at 20,000 × *g*. The insoluble fraction was acetone precipitated at –20 °C and digested in-gel with 200 ng modified trypsin (sequencing grade, Promega) for 18 hours at 37 °C. Resulting peptides were analyzed by LC-MS/MS on an Orbitrap-XL mass spectrometer (Thermo Scientific, Waltham, MA). Proteins were identified by database searching of the fragment spectra against the SwissProt (EBI) protein database using Mascot (v 2.3, Matrix Science, London, UK).

### Cellular oxygen consumption and extracellular acidification rate

The XF96 extracellular flux analyzer (Seahorse Biosciences) measured oxygen consumption rate (OCR) and extracellular acidification rate (ECAR) in a 96-well format. Before measurement, 30,000 cells per well were seeded and incubated for 16 h. Mitochondrial or glycolytic stress tests were performed as per the manufacturer’s instructions. NucBlue staining was utilized for cell counting and analysis of oxygen consumption and extracellular acidification rate.

### Circular dichroism (CD) spectroscopy

CD spectra of G3BP1 protein, annealed PCSK9 rG4 were recorded in RNase-free buffers (10 mM Tris HCl, pH 7.5, 100 mM KCl, 1 mM EDTA) within the wavelength range of 200–320 nm. Additionally, a mixture of 3 μM annealed PCSK9 rG4 and G3BP1 protein was incubated in the same buffer at 4 °C for 30 min. The CD spectrum of the G3BP1 protein was subtracted from the complex spectrum to obtain the CD spectrum of rG4 in the G3BP1 rG4 complex.

### Transwell assay

Tumor cell migration was assessed using Transwell chambers. Briefly, 5 × 10^4^ transfected TNBC cells in serum-free medium were seeded in the upper chamber, with full culture medium in the lower chamber. After 24 h, migrated cells were counted under a microscope.

### Colony formation assay

TNBC cells before and after treatment were plated at 500 cells per well in 6-well plates and cultured in RPMI 1640 medium supplemented with 10% FBS for two weeks. Colonies were fixed, stained with methanol and crystal violet, and counted.

### Luciferase reporter assay

The PCSK9 promoter or G3BP1 promoter was sub-cloned into the pGL3 dual-luciferase vector to generate luciferase reporter constructs. These constructs were transfected into MDA-MB-231/3T3-L1 cells, and luciferase activity was measured using the Dual-Luciferase Reporter Assay System (Promega).

### Co-immunoprecipitation (Co-IP)

In total, 400 μg of whole-cell lysates (WCL) were incubated overnight with 3 μg of anti-Flag (ab205606) or anti-HA antibody (ab9110), anti-CD86 (ab239075), anti-CD80 (ab134120), anti-Pan-Kac (#9441, Cell Signaling Technology, Beverly, MA, USA), or IgG control antibody in TBS buffer containing 0.4% NP-40. Specific cell lysates were incubated with primary antibodies and protein A/G magnetic beads overnight at 4 °C. After washing with lysis buffer, proteins were analyzed by western blotting with the corresponding antibodies.

### RNA pulldown

In total, 1.5 × 10^7^ MDA-MB-231/3T3-L1 cells were lysed in 500 μL IP buffer and incubated with Biotin-labeled probes for 2 h at room temperature. Streptavidin C1 magnetic beads (Invitrogen) were added for an additional hour of incubation. Beads were washed five times and subjected to Western blot analysis.

### Immunofluorescence (IF)

Cells were fixed with formaldehyde, permeabilized with Triton X-100, and blocked with 5% BSA at 4 °C overnight. Samples were incubated with primary antibodies against G3BP1 (ab56574, Abcam), rG4 (ab169652, Abcam), CD69 (ab322534, Abcam), CD25 (ab231441, Abcam), CD38 (ab235118, Abcam) or HLA-DR (ab20181, Abcam) for 1 h at 37 °C, followed by incubation with Alexa Fluor® 488-conjugated secondary antibody (ab150113 or ab150080 as needed; 1/1000 dilution). DAPI staining was performed, and fluorescence microscopy was conducted for visualization.

### Fluorescence anisotropy

Following protocols described by Hauser et al. [32], measurements were performed at 30 °C using a Synergy 2, Synergy 4, and Cytation 5 multimode plate reader with a black 96-well F-bottom plate. Fluorescence anisotropy was measured at an excitation wavelength of 540 nm and an emission wavelength of 620 nm using Gen 5 software. Data were analyzed using regression analysis to provide mean and SEM values.

### Hematoxylin and eosin (HE) staining

Tissues were fixed in 10% formalin and paraffin-embedded. Sections (5 μm thick) were stained with hematoxylin and eosin following standard protocols and examined under a light microscope.

### Immunohistochemistry (IHC)

Tumor tissue samples are stained by IHC using PCSK9 (55206-1-AP, Proteintech), CD86 (ab220188, Abcam), or Ki67 (ab15580, Abcam) antibodies according to the manufacturer’s protocol. Tumor tissue sections were dewaxed and subsequently rehydrated. The antigen was immersed in a pH 6.0 citrate buffer at 95 °C for 15 min. Subsequently, the endogenous peroxidase activity was blocked by incubating the slices with 0.3% hydrogen peroxide for 15 min at room temperature. Eventually, the antigen is recycled. We then closed sections with PBS rinse and 5% normal goat serum at room temperature for 30 min before applying the monoantibody at 4 °C overnight. After TBST washing, the slices were incubated with secondary antibodies at 37 °C for 1 h. DAB is used for color development, and hematoxylin is used for nuclear restaining.

### Statistical analysis

Statistical analyses were conducted using GraphPad Prism. The experiments were performed independently three times, with three biological replicates per sample. Data were expressed as mean ± standard deviation (SD), excluding those not meet normal distribution according to the pre-established criteria. Two-tailed Student’s *t* tests were employed for comparisons between two groups, while one-way analysis of variance (ANOVA) followed by Tukey’s multiple comparisons test was used for comparisons among multiple groups. The overall survival of TNBC patients was shown as a Kaplan-Meier curve. Statistical significance was defined as *P* < 0.05. Data analyzer were blinded to the experimental conditions.

## Results

### TNBCs promote lipolysis in adipocytes leading to the formation of cancer-associated adipocytes

**Fig. 1:**
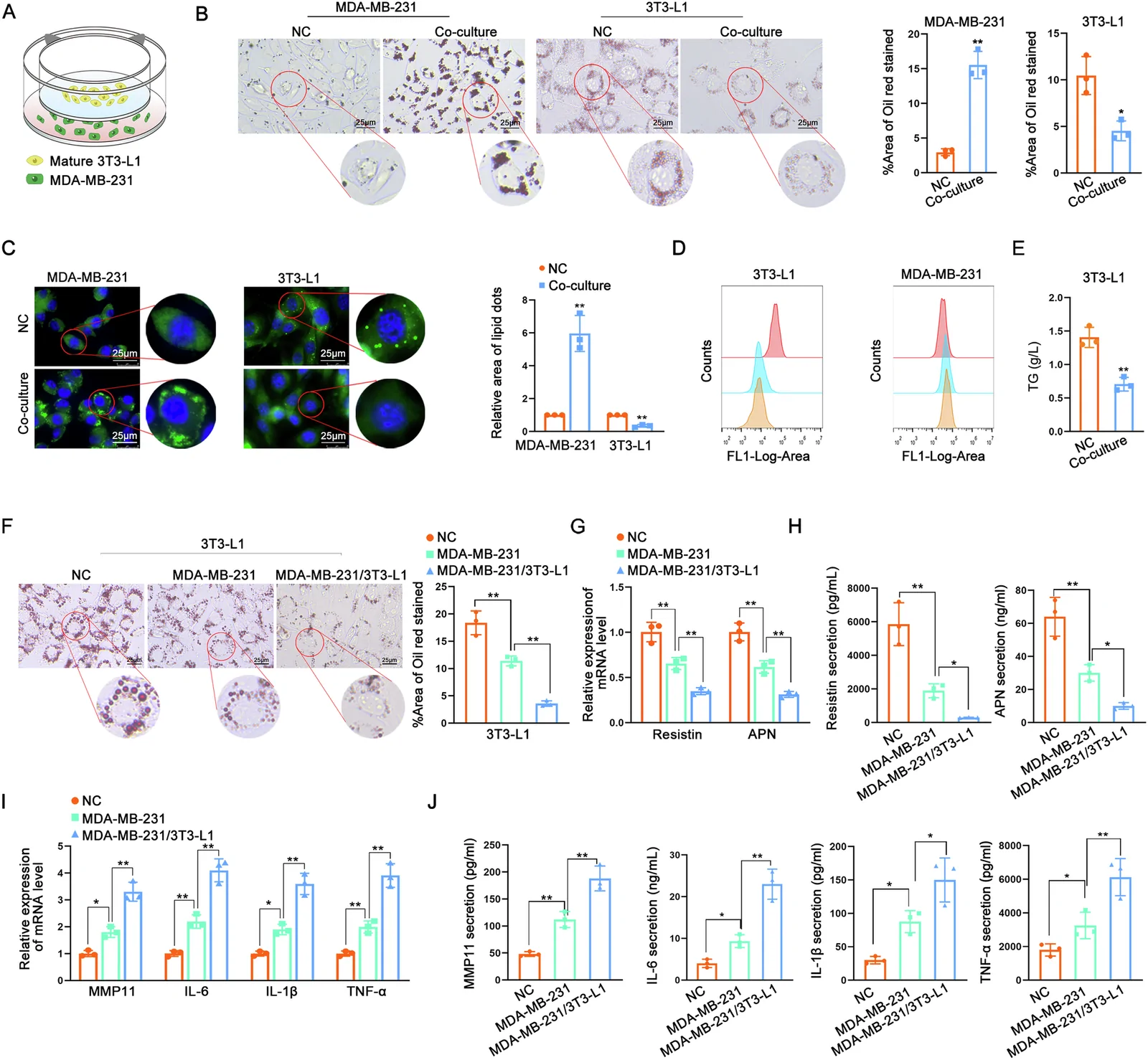
TNBCs promote lipolysis in adipocytes leading to the formation of tumor-associated adipocytes. **A** Schematic representation of the co-culture model of adipocytes and TNBC cells. **B** Oil Red O staining quantifying lipid content in adipocytes and TNBC cells. **C** Bodipy staining detecting lipid content in adipocytes and TNBC cells. **D** Flow cytometric analysis of lipid droplet uptake by TNBC cells from adipocytes. **E** Quantification of triglyceride content in adipocytes. **F** Oil Red O staining of adipocytes treated with tumor cells influenced or not influenced by adipocytes. **G** RT-qPCR analysis of Resistin and APN in adipocytes treated with tumor cells, influenced or not influenced by adipocytes. **H** ELISA analysis of Resistin and APN in adipocytes treated with tumor cells, influenced or not influenced by adipocytes. **I** RT-qPCR analysis of MMP11, IL-6, IL-1β, and TNF-α in adipocytes treated with tumor cells, influenced or not influenced by adipocytes. **J** ELISA analysis of MMP11, IL-6, IL-1β, and TNF-α in adipocytes treated with tumor cells, influenced or not influenced by adipocytes. Statistical significance: ^*^P < *0.05*, ^**^P < *0.01*.

To assess the phenotypic changes in both adipocytes and TNBC cells and to investigate the key regulators that facilitate their crosstalk, we established a co-culture system of 3T3-L1 adipocytes and TNBC cells (Fig. 1A). After 48 h of co-culture, we conducted Oil Red O staining and Bodipy C_16_ staining, observing a greater than 3-fold increase in lipid accumulation within the tumor cells, along with a reduction of over 50% in both the number and size of lipid droplets in adipocytes (Fig. 1B–D). And triglyceride (TG) accumulation in adipocytes also decreased by more than 50% after co-culture with tumor cells (Fig. 1E). These findings suggest that co-culture leads to lipolysis in adipocytes, which, in turn, increases lipid uptake by tumor cells. More strikingly, lipolysis in adipocytes was further enhanced when co-cultured with tumor cells that had previously been exposed to adipocytes (Fig. 1F). This observation indicated that these tumor cells may accumulate the ability to exacerbate metabolic dysregulation associated with obesity and cancer.

Previous studies have shown that tumor cells induce adipocyte reprogramming into CAA, supporting tumor progression [18–20]. In the current study, we observed that terminal differentiation markers Resistin and APN were significantly decreased in the adipocytes upon exposure to tumor cells, and even more so when exposed to adipocyte-educated tumor cells (Fig. 1G, H). Conversely, CAA markers, including MMP11, IL-6, IL-1β, and TNF-α, were significantly upregulated, with a further increase observed in adipocytes exposed to tumor cells preconditioned by adipocytes (Fig. 1I–J). Interestingly, data from Kaplan-Meier plotter indicated that TNBC samples with higher levels of CAA markers like IL-6 and MMP11 indicated poorer prognoses of the patients (Fig. S1A-B). This implied that factors affecting CAA formation might be targets for TNBC therapy.

### Tumor cells-derived extracellular vesicles transfer PCSK9 to adipocytes, converting them into CAAs

**Fig. 2:**
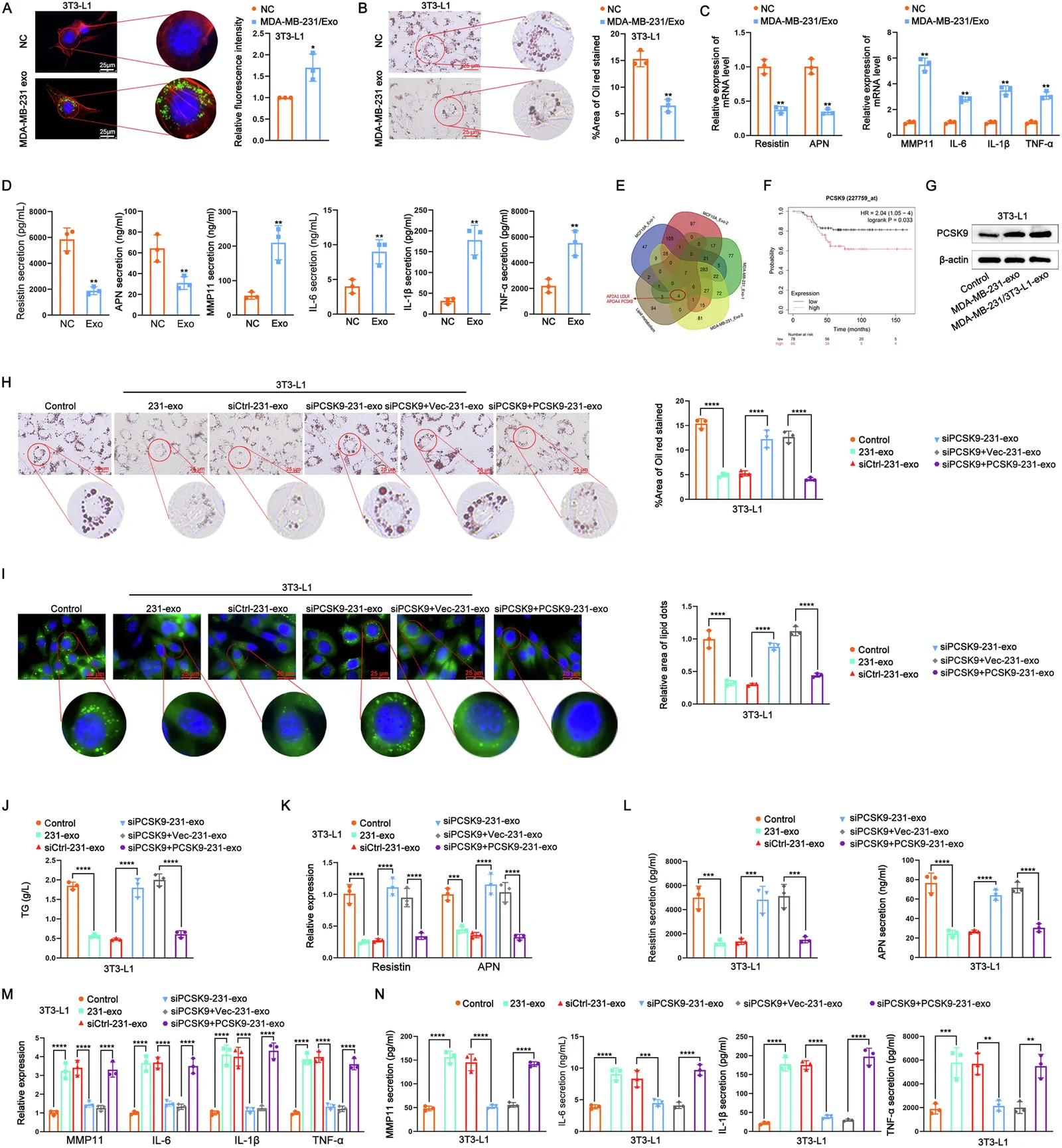
Tumor cells induce lipolysis in adipocytes to form tumor-associated adipocyte phenotypes through the delivery of PCSK9 via exosomes. **A** Exosome tracer experiment to track exosome uptake by adipocytes. **B** Oil Red O staining of adipocytes treated with tumor-derived exosomes. **C** RT-qPCR analysis of terminal differentiation marker expression in adipocytes treated with tumor-derived exosomes. **D** ELISA analysis of tumor-associated adipocyte marker expression in adipocytes treated with tumor-derived exosomes. **E** Exosomes LC/MS-based proteomic analysis of normal breast epithelial cells and TNBC cells. **F** Kaplan-Meier plotter identified the role of PCSK9 in TNBC. **G** Western blot analysis of PCSK9 content in adipocytes treated with exosomes from TNBC cells co-cultured with or without adipocytes. **H** Oil Red O staining of adipocytes treated with exosomes from the indicated TNBC cells. **I** Bodipy staining of adipocytes treated with exosomes from TNBC cells under different conditions. **J** Triglyceride content in adipocytes after treatment with exosomes from TNBC cells under different conditions. **K** RT-qPCR analysis of Resistin and APN in adipocytes after treating exosomes from TNBC cells under different conditions. **L** ELISA analysis of Resistin and APN in adipocytes with exosome treatment from TNBC cells under different conditions. **M** RT-qPCR analysis of MMP11, IL-6, IL-1β, and TNF-α in adipocytes with exosome treatment from indicated TNBC cells. **N** ELISA analysis of MMP11, IL-6, IL-1β, and TNF-α in adipocytes after treating exosomes from TNBC cells under different conditions. Statistical significance: ^**^P < 0.01.

To elucidate the key molecular mechanisms driving reciprocal communication between adipocytes and tumor cells, we focused on analyzing the content of tumor-associated extracellular vesicles (EVs), which have emerged as crucial mediators of intercellular communication that promote tumor progression [33–35]. We purified tumor cell-secreted EVs that exhibited the expected exosome size distribution, ranging from 110 to 150 nm in diameter (Fig. S2A-B). The isolation of exosomes was confirmed by the presence of exosome marker proteins, including CD63 and Hsp70, and the absence of calnexin, an endoplasmic reticulum-specific protein (Fig. S2C). Next, using an in vitro EV tracing assay, we demonstrated that adipocytes were capable of taking up tumor-derived exosomes efficiently, evidenced by the presence of green fluorescence staining in these cells (Fig. 2A). Thus, our results confirmed that exosomes facilitate cellular communication between adipocytes and TNBC cells.

We next sought to confirm whether tumor-derived exosomes induce lipolysis in adipocytes and promote their transformation into CAAs. Treatment of adipocytes with exosomes derived from MDA-MB-231 cells resulted in a significant reduction in lipid droplet accumulation, reprogramming them into CAAs. This was evidenced by a decrease in adipocyte differentiation markers such as Resistin and APN, alongside an increase in CAA markers, including MMP11, IL6, IL-1β, and TNF-α (Fig. 2B–D).

To identify the EV cargos responsible for CAA induction, we performed the LC/MS-based proteomics on exosomes derived from normal breast epithelial cells (MCF10A) and TNBC cells (MDA-MB-231). Four proteins associated with lipid digestion and mobilization were selectively identified in MDA-MB-231-derived EVs, but not in those derived from normal breast epithelial cells (PathCards, https://pathcards.genecards.org/) (Fig. 2E). Previous studies have demonstrated that PCSK9 inhibition suppresses tumor growth and enhances tumor response to immune checkpoint therapy [36–45]. Importantly, TNBC patients with higher PCSK9 expression have significantly poorer prognoses than those with low PCSK9 expression (Fig. 2F). Furthermore, we found that treatment of 3T3-L1 adipocytes with either TNBC-derived exosomes or adipocyte-pre-educated TNBC-derived exosomes increased PCSK9 levels (Fig. 2G). Hence, we suspected that PCSK9 was transferred from TNBC cells to adipocytes via exosomes.

We aimed to determine the function of PCSK9 in adipocyte-tumor cell communication. We observed that PCSK9 knockdown by si-PCSK9 led to a marked decrease in both PCSK9 mRNA and protein levels; however, both levels recovered when co-transfected with PCSK9 overexpression vectors (Fig. S2D-E). Furthermore, the PCSK9 level was significantly reduced in the exosomes isolated from PCSK9 knockdown MDA-MB-231 cells, while exosomal PCSK9 level was then normalized under further PCSK9 overexpression (Fig. S2F). Next, exosomes carrying varying levels of PCSK9 from TNBC cells were applied to adipocytes. Results showed that the size and number of lipid droplets and TG abundance in adipocytes declined after treatment with exosomes from MDA-MB-231 cells, but recovered when treated with exosomes from MDA-MB-231 cells under PCSK9 knockdown, while re-declined when the PCSK9 level was normalized in TNBC cell-derived exosomes (Fig. 2H–J). More notably, the conversion of adipocytes into CAAs by tumor-derived exosomes was reversed by PCSK9 loss but persisted after PCSK9 recovery in these exosomes. This was demonstrated by changes in the expression and secretion of adipocyte terminal differentiation markers such as Resistin and APN (Fig. 2K, L), as well as changes in CAA markers, including MMP11, IL6, IL-1β, and TNF-α (Fig. 2M, N). Taken together, these results reinforced the central role of PCSK9 in tumor cell-adipocyte communication and in facilitating the transformation of the CAA phenotype.

### Adipocytes drive lipid metabolic reprogramming in TNBC cells, promoting tumor progression

**Fig. 3:**
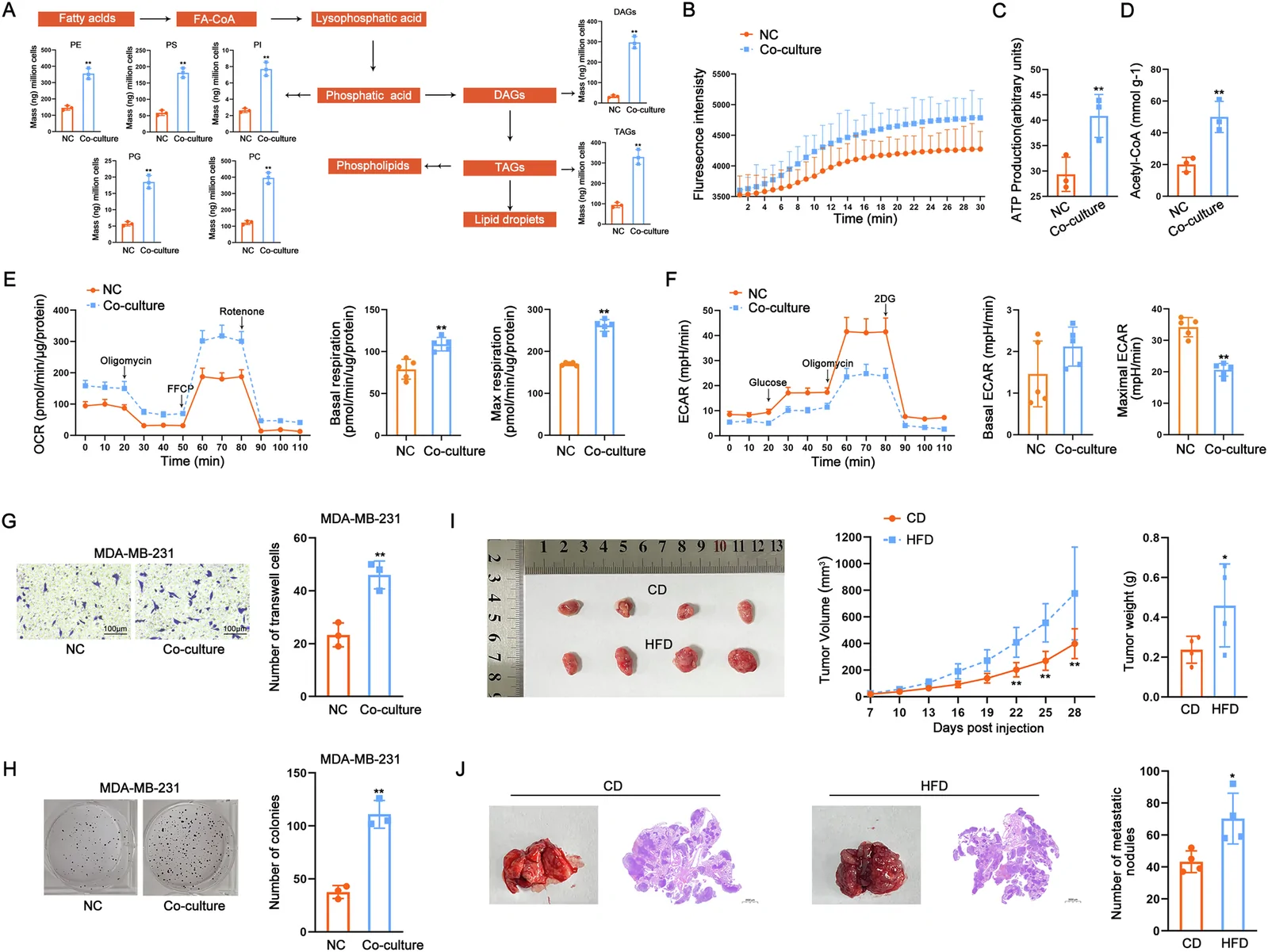
Lipid uptake by tumor cells promotes metabolic changes. **A** Liquid chromatography-tandem mass spectrometry (LC-MS/MS) analysis of downstream metabolites of fatty acids, including lipid species: PI (phosphatidylinositols), PG (phosphatidylglycerols), PS (phosphatidylserines), PE (phosphatidylethanolamines), and PC (phosphatidylcholines). **B** FAO levels in TNBC cells co-cultured with or without adipocytes. **C** ELISA determination of ATP content in TNBC cells co-cultured with or without adipocytes. **D** ELISA determination of acetyl-CoA content in TNBC cells co-cultured with or without adipocytes. **E** Seahorse XF analysis of OCR in TNBC cells co-cultured with or without adipocytes. **F** Seahorse XF analysis of ECAR in TNBC cells co-cultured with or without adipocytes. **G** Transwell migration assay of TNBC cells co-cultured with or without adipocytes. **H** Colony formation assay to analyze TNBC cell proliferation co-cultured with or without adipocytes. **I** Tumor gross, growth volume, and weight in different diet groups. **J**. HE staining of metastatic nodules in the lungs of mice from different diet groups. Statistical significance: ^**^P < 0.01.

Reciprocally, adipocyte-TNBC cell coculture resulted in notable lipid droplet accumulation within tumor cells, as illustrated in Fig. 1B, C. To gain insight into how the adipocytic microenvironment reprograms lipid metabolism in TNBC cells, we employed liquid chromatography-tandem mass spectrometry (LC-MS/MS) to analyze changes in lipid mediators derived from fatty acids in tumor cells influenced by adipocytes (Fig. 3A). This co-culture led to a significant increase in the levels of PI (phosphatidylinositols), PG (phosphatidylglycerols), PS (phosphatidylserines), PE (phosphatidylethanolamines), and PC (phosphatidylcholines), Diacylglycerols (DAG), and Triacylglycerols (TAG) in MDA-MB-231 cells (Fig. 3A). Moreover, co-culture with adipocytes exhibited increased fatty acid β-oxidation (FAO) in TNBC cells, along with increased ATP production and acetyl-CoA generation (Fig. 3B–D). We used the Seahorse cell metabolic dynamic analysis system to assess FAO status in TNBC cells with or without adipocyte exposure. The results showed a significantly increased OCR in co-cultured TNBC cells, both in basal and maximal-uncoupled states (Fig. 3E). Concurrently, a decrease in ECAR was observed in TNBC cells after co-culture with adipocytes, indicating that adipocyte interaction disrupted glycolysis in tumor cells (Fig. 3F). These findings suggested that adipocytes reshape the fatty acid energy metabolism pathway, facilitating a shift in tumor cells from glycolysis to the more energy-efficient FAO pathway.

Functionally, adipocyte-mediated metabolic reprogramming is associated with enhanced tumor aggressiveness. Our data indicated that adipocytes promoted migration and proliferation of MDA-MB-231 cells (Fig. 3G, H). To further validate the role of an adipocyte-rich microenvironment in promoting the progression of TNBC in vivo, we fed female BALB/c mice with a high-fat diet (HFD) or a regular chow diet (CD) for 12 weeks. Subsequently, we established an orthotopic allograft model by transplanting a luciferase-labeled TNBC cell line, 4T1, into the mammary fat pad, and an experimental metastatic model by tail vein injection of 4T1 cells. The HFD resulted in prominent obese phenotypes, with significant increases in both body weight and fat mass (Fig. S3A). The HFD impaired glucose intolerance in mice, as demonstrated by glucose tolerance tests (GTT), and activated FAO, as reflected by elevated serum FAO levels (Fig. S3B-C). HFD-induced obesity significantly accelerated tumor growth and metastasis (Fig. 3I–J). In addition, tumors in the HFD group showed increased Ki67 expression (Fig. S3D). Collectively, these findings illustrate the impact of an HFD on the malignant phenotype of TNBC and underscore the critical role of adipocytes in TNBC progression.

### Adipocytes enhance FAO in tumor cells to stabilize PCSK9 via a G3BP1-PCSK9 rG4 interaction

**Fig. 4:**
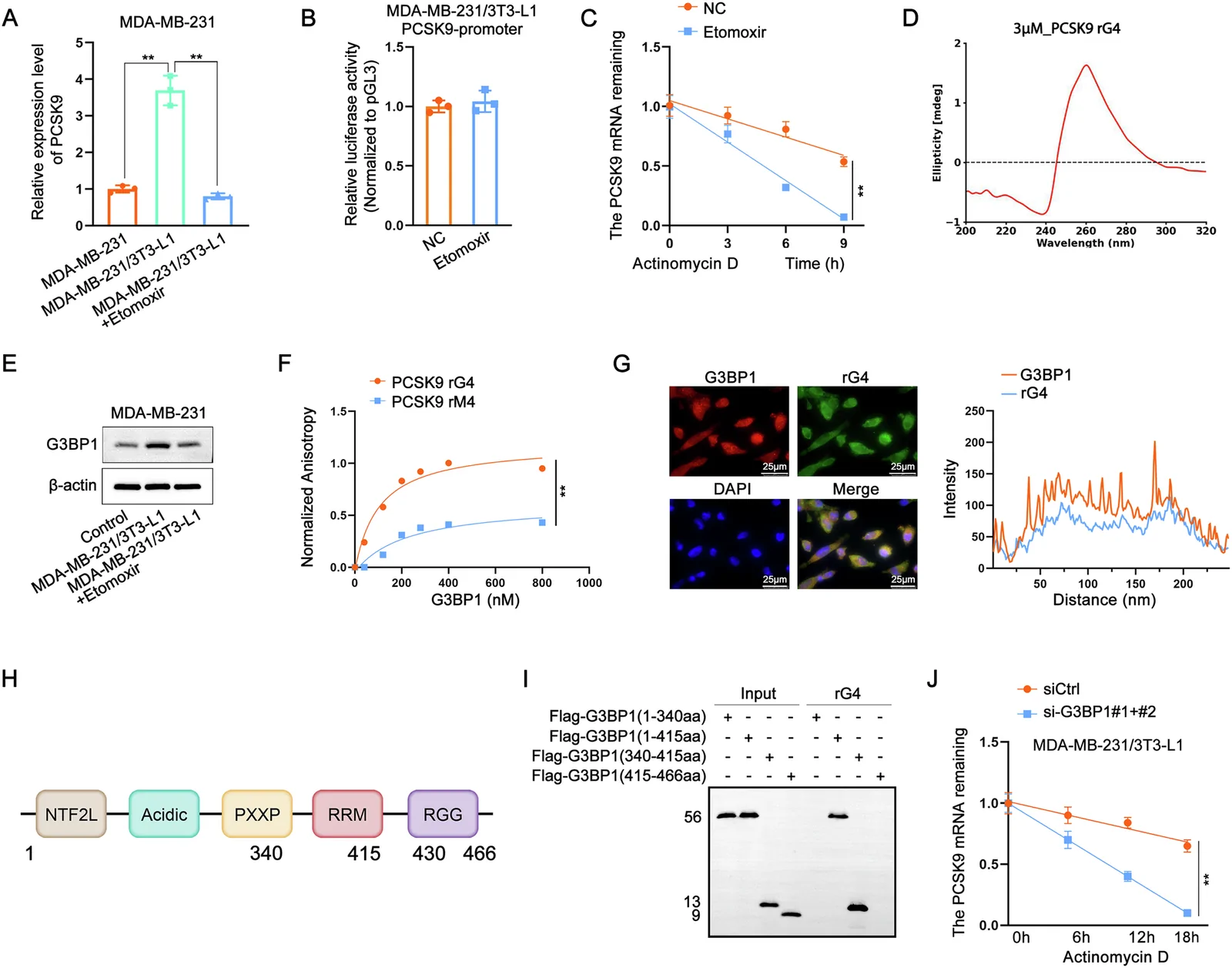
G3BP1 regulates the stability of PCSK9 through recognizing rG4 structures. **A** RT-qPCR analysis of PCSK9 mRNA levels in TNBC cells co-cultured with adipocytes or adipocytes plus FAO inhibitor (Etomoxir). **B** Luciferase reporter assay to evaluate the impact of FAO on PCSK9 promoter activity. **C** RT-qPCR analysis of FAO impact on PCSK9 mRNA stability. **D** Circular dichroism spectroscopy to analyze PCSK9 structural changes. **E** Western blot analysis of PCSK9 levels in TNBC cells co-cultured with adipocytes or adipocytes plus Etomoxir. **F** Fluorescence anisotropy to identify G3BP1 binding domains. **G** Immunofluorescence analysis of rG4 and G3BP1 co-localization. **H** Schematic representation of G3BP1 structure. **I** RNA pull-down assay to identify the G3BP1 domain that recognizes the rG4 structure in PCSK9 mRNA. **J** RT-qPCR analysis of PCSK9 mRNA stability after G3BP1 interference. Statistical significance: ^**^P < 0.01.

Considering that adipocyte-educated TNBC cells have stronger induction for adipocyte-CAA transformation than parental TNBC cells (Fig. 1F–J), we then questioned whether adipocytes function as feedback regulators for PCSK9 expression in tumor cells via the upregulation of FAO. Our results demonstrated that the PCSK9 level in tumor cells increased upon exposure to adipocytes. Notably, the inhibition of FAO with Etomoxir eliminated this upregulation (Fig. 4A). These findings suggested that adipocytes influence FAO in tumor cells to regulate PCSK9 expression. Mechanistically, we found that the FAO inhibitor Etomoxir significantly shortened the half-life of PCSK9 mRNA in tumor cells without affecting its promoter activity, as shown in the luciferase reporter assay (Fig. 4B, C). These results suggested that co-culturing tumor cells with adipocytes enhances the stability of PCSK9 mRNA, possibly by upregulating FAO. Recently, RNA guanine quadruplexes (rG4s), which are prevalent in the 5’ and 3’ untranslated regions (UTRs) of mRNA, have been implicated in modulating post-transcriptional regulation, controlling mRNA targeting, processing, translation, and degradation [46–48]. Interestingly, we conducted G-quadruplex structure prediction of PCSK9 mRNA using QGRS Mapper and identified numerous rG4 structures within its 3’ UTR (Fig. S4A). Circular dichroism (CD) spectroscopy revealed that PCSK9 rG4 sequences can assemble into parallel G4 topological structures (Fig. 4D). Hence, we wondered whether the G-quadruplex structure of PCSK9 mRNA could influence its stability.

The important roles of rG4 in regulating RNA metabolism are often modulated by rG4-binding proteins. Based on the proteomic profiles of exosomes from normal breast cells and TNBC cells, we identified that G3BP1, which has been shown to positively regulate mRNA stability via its binding to rG4 structures [49]. Consistently, our results showed a significant increase in G3BP1 protein levels in TNBC cells after adipocyte treatment, which was reversed by the FAO inhibitor Etomoxir (Fig. 4E). Previously, FAO was suggested to contribute to NF-κB activation by inducing RelA acetylation [50], and NF-κB inhibition suppresses TNF-α-induced G3BP1 expression in breast cancer cells [51]. Hence, we hypothesized that adipocyte coculture-induced elevated FAO may activate the NF-κB pathway to facilitate G3BP1 expression in TNBC cells. It showed that adipocyte coculture increased acetylated RelA levels, which were normalized by FAO inhibition with Etomoxir (Fig. S4B). As a result, NF-κB activity was increased in MDA-MB-231 cells after 3T3-L1 coculture, but was reversed by Etomoxir treatment (Fig. S4C). Furthermore, 3T3-L1 coculture-boosted G3BP1 level was normalized under NF-κB inhibition by BAY 11-7082 (Fig. S4D). Altogether, adipocytes enhance FAO in TNBC cells to activate NF-κB to elevate G3BP1 expression.

We then assessed the binding capacity of G3BP1 to PCSK9 rG4 probes and to the corresponding mutated probes (rM4), which are unable to fold into the G4 structure. The fluorescence anisotropy measurements showed that G3BP1 bound strongly to PCSK9 rG4 probes (Fig. 4F). Immunofluorescence staining further confirmed the G3BP1-rG4 interaction (Fig. 4G). G3BP1 is a conserved, multi-domain protein comprising an N-terminal NTF2-like domain, an acidic-rich region, PXXP motifs, an RNA recognition motif (RRM), and a C-terminal arginine-glycine-glycine (RGG) domain. The RRM and RGG domains are RNA-binding domains [49, 52]. We generated several truncated forms of the G3BP1 protein and found that the RRM domain of G3BP1 specifically recognized the rG4 structure of PCSK9 mRNA, but not the corresponding mutated structure rM4 (Fig. 4H–I, Fig. S4E). To demonstrate the functional relevance of G3BP1-PCSK9 rG4 interaction, we knocked down G3BP1 in MDA-MB-231 cells and found that the half-life of PCSK9 mRNA was significantly shortened in G3BP1 knockdown tumor cells upon exposure to adipocytes (Fig. 4J, Fig. S4F). Moreover, PCSK9 protein levels in these cells and their derived exosomes were both decreased (Fig. S4G). Taken together, our results emphasize that co-culturing tumor cells with adipocytes increases G3BP1 expression, stabilizing PCSK9 mRNA via the G3BP1-PCSK9 rG4 interaction, thereby maintaining high PCSK9 protein levels in tumor cells.

### CAAs enhance tumor progression by impeding T cell activation

**Fig. 5:**
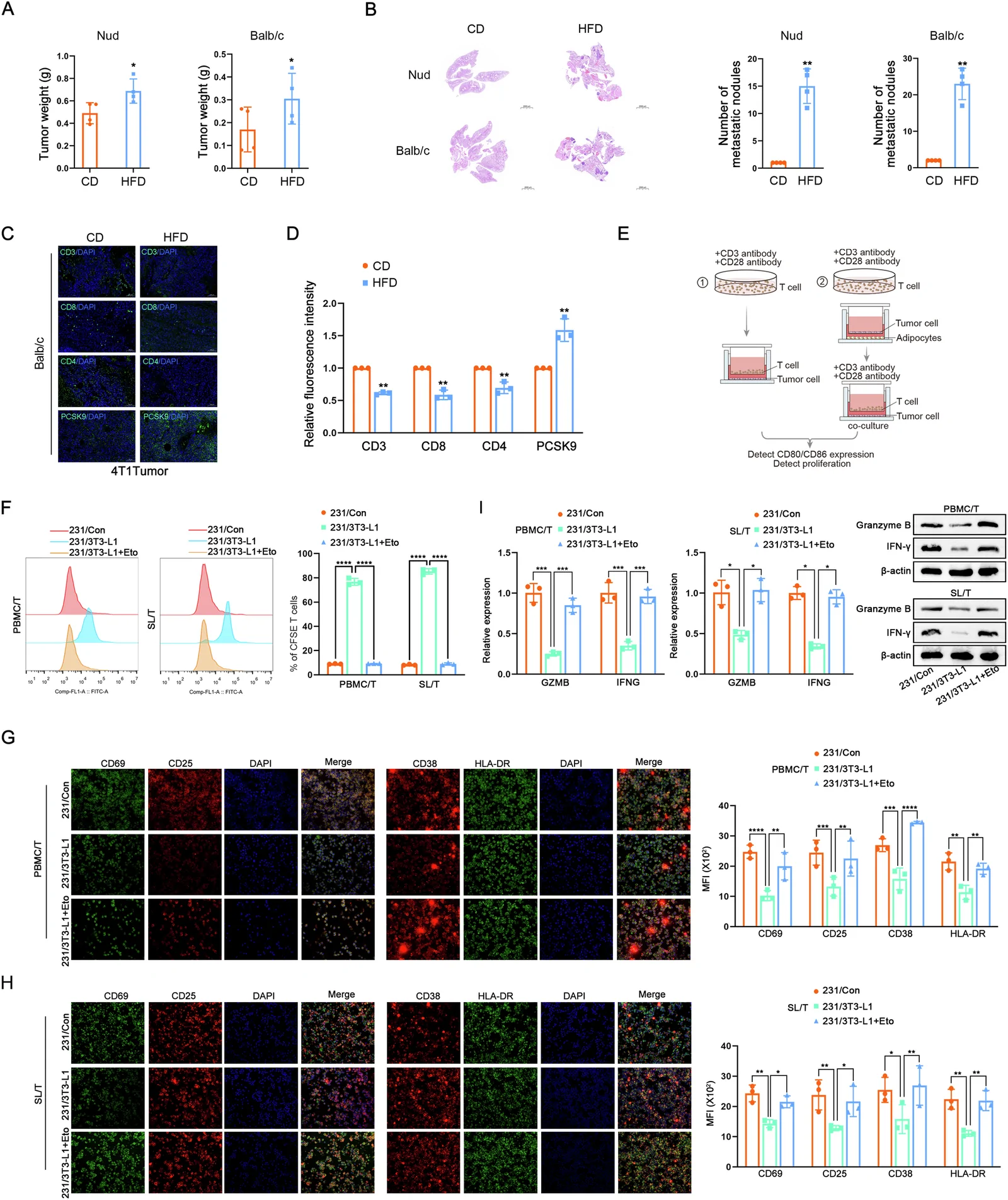
High-fat diet promotes breast cancer metastasis. **A** Tumor weight of breast cancer models in nude and Balb/c mice. **B** HE staining of lung metastases in breast cancer models of nude and Balb/c mice. **C** Immunofluorescence analysis of CD3, CD4, CD8, and PCSK9 expression in tumor tissues from breast cancer models established in Balb/c mice. **D** Correlation analysis of PCSK9 expression and immunoinfiltration in the above tumors. **E** A schematic diagram illustrating T cell activation under different stimuli by TNBC cells with or without adipocyte co-culture. **F** Flow cytometric analysis of T cell proliferation impacted by TNBC cells with or without adipocyte co-culture. **G**, **H** Immunofluorescence analysis of CD69, CD25, CD38, and HLA-DR expression in T cells stimulated by the indicated TNBC cells. **I** RT-qPCR and western blot analyses revealed changes in the expression of T cell effectors, including GZMB/Granzyme B and IFNG/IFN-γ, in T cells stimulated by the indicated TNBC cells. Statistical significance: ^*^P < 0.05, ^**^P < 0.01.

Previous studies have shown that tumor-induced changes to adipocytes, as CAAs, are positively associated with an immunosuppressive microenvironment in TNBC patients [25, 26, 53]. To validate this phenotype, we first established HFD-induced obese nude mice and Balb/c mice and injected breast cancer cells (4T1) into the mammary fat pads of these mice to monitor the primary breast tumor progression and lung metastasis. Notably, HFD accelerated primary tumor growth and lung metastasis in both nude mice and BALB/c mice, with an even more pronounced enhancement observed in BALB/c mice (Fig. 5A, B). These results highlighted the potential roles of the immune system in mediating tumor progression and metastasis through adipocytes.

Furthermore, PCSK9 has been identified as a prognostic and immune-related influencing factor in tumorigenesis, and its inhibition has been shown to potentiate immune checkpoint therapy for cancer [54–59]. Based on TIMER (tumor immune estimation) analysis of basal-like breast cancer samples in the TCGA dataset, we found a significant negative correlation between PCSK9 expression and CD8^+^ T-cell infiltration levels (Fig. S5). Additionally, tumors in HFD-induced obese mice exhibited significantly reduced lymphocytic infiltrates compared with those in the control group, and these infiltrates were negatively correlated with PCSK9 expression (Fig. 5C, D).

We then aimed to determine whether these CAAs facilitate tumor immune escape by modulating T-cell activation. We co-cultured tumor cells, either treated or untreated, with adipocytes for 24 h, with T cells isolated from human peripheral blood mononuclear cells (PBMCs) or spleen lymphocytes (SLs) from C57BL/6 mice after stimulation with anti-CD3/CD28 antibodies and IL2 (Fig. 5E). Further, to determine whether the effects of CAAs on T cells depended solely on lipid load or on FAO in TNBC cells, a commonly used FAO inhibitor, Etomoxir, was applied. Notably, adipocyte-educated tumor cells significantly suppressed the proliferation of both T cells from PBMCs and SLs compared to naïve tumor cells. In contrast, this suppression was reversed upon FAO inhibition with Etomoxir (Fig. 5F). Furthermore, the fluorescence intensities of T-cell activation markers, including CD69, CD25, CD38, and HLA-DR, were significantly decreased in T cells cocultured with adipocyte-educated tumor cells, and all the signals were recovered upon Etomoxir treatment (Fig. 5G, H). Moreover, the levels of T cell effectors, including GZMB/Granzyme B and IFNG/IFN-γ, were reduced in the face of MDA-MB-231 cells under adipocyte coculture, but regained after FAO inhibition (Fig. 5I). In conclusion, adipocytes co-cultured with TNBC cells hamper T cell activation by facilitating FAO. Nevertheless, the detailed mechanisms underlying adipocyte-educated TNBC cells’ mediated suppression of T-cell activation remain to be uncovered.

### Adipocytes boost FAO in tumor cells to promote autophagic degradation of T cell costimulatory molecules CD80/CD86, dampening T cell activation

**Fig. 6:**
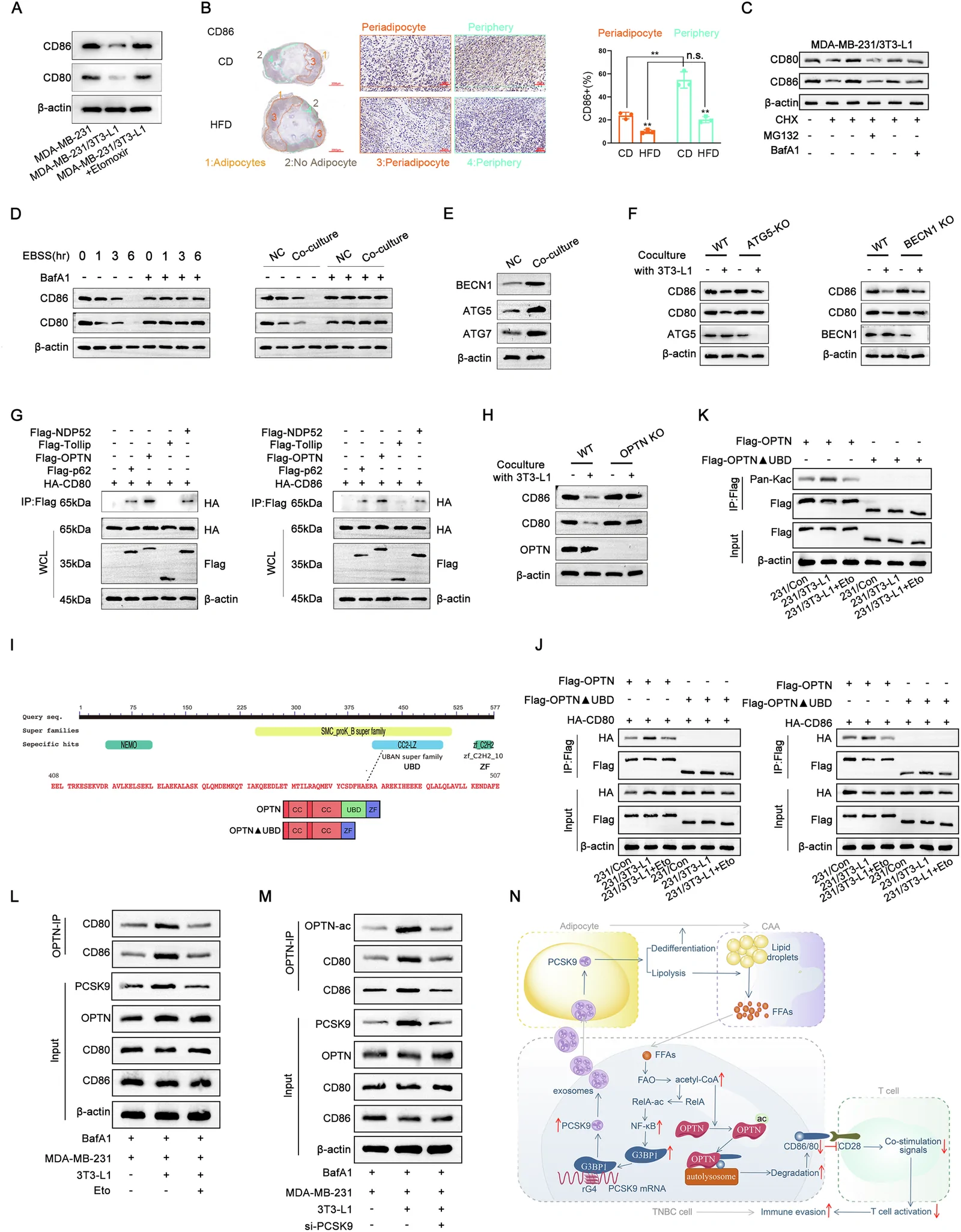
The autophagic degradation of CD80/CD86 in TNBC cells mediated via the PCSK9-FAO-OPTN axis. **A** Western blot analysis of CD80 and CD86 expression in adipocytes co-cultured with tumor cells or together with FAO inhibition. **B** IHC was used to detect the expression of CD86 in tumor tissues from the standard diet group and the high-fat diet group. **C** Western blot analysis of CD80 and CD86 levels in adipocyte-educated TNBC cells under different treatments. **D** Western blot confirmation of CD80 and CD86 degradation through autophagy in co-cultured cells. **E** Western blot analysis of autophagy-related proteins in co-cultured cells. **F** Western blot confirmation that inhibition of autophagy increases CD80 and CD86 expression. **G** Co-immunoprecipitation (Co-IP) analysis of the binding of different autophagic receptors to CD80 and CD86. **H** Western blot analysis showing that OPTN knockout promotes CD80 and CD86 expression. **I** Schematic of OPTN structure and mutation sites. **J** Co-IP analysis of the interaction of UBD-mutated or full-length OPTN with CD80 and CD86 in indicated TNBC cells. **K** Co-IP assessed the acetylation of UBD-mutated or full-length OPTN in different TNBC cells. **L** Co-IP examined the binding between OPTN and CD80/CD86 in different TNBC cells. **M** Co-IP examined the acetylation of OPTN and its binding with CD80/CD86 in different TNBC cells. **N** The schematic summarization of the PCSK9-CAA-FAO-OPTN axis responsible for the communications among adipocytes, TNBC cells, and T cells. *Statistical significance:* ^**^P < 0.01; n.s., not significant.

Co-stimulation is essential for T cell activation. The interaction between CD80 and/or CD86 on the antigen-presenting cells with CD28 and/or CTLA4 on T cells is required to trigger T-cell activation [60]. It meant that CD80/CD86 expression in TNBC cells largely correlated with T cell activation and their recognition of TNBC cells. Hence, we focused on the changes in CD80/CD86 expression in TNBC cells. After co-culturing tumor cells with adipocytes, we found that both CD80 and CD86 levels were significantly reduced; however, this phenotype was restored by FAO inhibition with Etomoxir (Fig. 6A). IHC staining demonstrated that tumor samples in the HFD group exhibited a dramatic decrease in CD86 expression, relative to those from the CD group. More strikingly, CD86 expression was lower in tumors near periadipocytes than in those at the periphery, and this was specifically observed in tumors from CD-fed mice (Fig. 6B).

To further investigate the pathways involved in CD80/CD86 protein turnover, we treated adipocyte-treated tumor cells with the protein synthesis inhibitor cycloheximide (CHX), the proteasome inhibitor MG132, or the lysosome inhibitor bafilomycin A1 (BafA1). Results indicated that CD80/CD86 levels were reduced under CHX treatment, suggesting that the proteins were degraded when synthesis was inhibited. However, only co-treatment with BafA1, but not MG132, significantly blocked the degradation of CD80/CD86, suggesting a possible role for the autophagy-lysosome pathway in the degradation of CD80/CD86 in adipocyte-educated TNBC cells (Fig. 6C). Further validation showed that co-culturing tumor cells with adipocytes led to a significant reduction in CD80/CD86 expression, which was completely reversed by BafA1 treatment. These results were consistent with the positive control, as tumor cells were treated with autophagy inducer Earle’s Balanced Salt Solution (EBSS) for the indicated times, in the presence or absence of BafA1 (Fig. 6D). We then compared the expression levels of key autophagy-related markers, including BECN1, ATG5, and ATG7, in tumor cells. All these proteins increased dramatically in adipocyte-treated tumor cells compared to tumor cells in monoculture conditions (Fig. 6E). Knockout of autophagy-related genes ATG5 or BECN1 partially restored CD86/CD80 expression in adipocyte-exposed tumor cells (Fig. 6F). The above data indicate that adipocytes induce autophagic degradation of CD80/CD86 in TNBC cells by facilitating FAO.

To identify the autophagic cargo receptors potentially mediating CD80/CD86 autophagic degradation, we generated tagged vectors for a panel of candidates, including CALCOCO2 (NDP52), Tollip, OPTN, and SQSTM1 (p62), using MDA-MB-231. Our analysis revealed that OPTN had the highest binding affinity for both CD80 and CD86, suggesting its role as the primary cargo receptor in this context (Fig. 6G). Adipocytes lost the capacity to induce CD80/CD86 degradation in OPTN-knockout tumor cells, further confirming the critical role of OPTN in adipocyte-mediated CD80/CD86 autophagic process (Fig. 6H). OPTN belongs to a group of ubiquitin-binding autophagy receptors. The domain structure of OPTN includes two coiled-coil domains, a canonical LIR motif, a ubiquitin-binding UBAN domain (UBD), and a C-terminal C2H2-type zinc finger (ZnF) (Fig. 6I) [61–63]. To explore the role of ubiquitination in OPTN-mediated CD80/CD86 degradation, we constructed a mutant vector that caused a deletion of the 408-507aa in the UBD domain (Fig. 6I). The results showed that Flag-OPTN^UBD^ lost the ability to bind to CD80/CD86; moreover, Flag-OPTN binding to CD80/CD86 was enhanced under adipocyte coculture, but this enhancement was abolished when Etomoxir was added (Fig. 6J). The data suggested that OPTN recognizes CD80/CD86 depending on its UBD, and that these bindings are regulated by FAO.

It is well known that the FAO process often generates acetyl-CoA, which serves as the acetyl donor for protein acetylation. Intriguingly, it has been revealed that acetylation of the UBD of autophagy receptors, such as p62 [64], can generally strengthen their affinity for ubiquitinated substrates [65]. Hence, we speculated that OPTN, which also harbors a UBD, might be acetylated, as is p62, to enhance its affinity for ubiquitinated substrates. As expected, only the acetylation level of Flag-OPTN was boosted in MDA-MB-231 cells with 3T3-L1 coculture, but recovered upon FAO inhibition with Etomoxir (Fig. 6K). Further, we found that although elevated PCSK9 expression in MDA-MB-231 cells under 3T3-L1 coculture was reversed by Etomoxir, the levels of OPTN and CD80/CD86 were not affected by adipocyte coculture or Etomoxir in BafA1-treated MDA-MB-231 cells. However, the interactions between OPTN and CD80/CD86 were strengthened in 3T3-L1-cocultured MDA-MB-231 cells, while the addition of Etomoxir normalized such phenomena (Fig. 6L). The above data strongly suggested that TNBC cells under adipocyte coculture contribute to OPTN acetylation, thereby enhancing its affinity for CD80/CD86 by aggravating FAO. In addition, given the essential role of PCSK9 in mediating adipocyte-tumor cell communication, we also assessed the impact of PCSK9 knockdown on OPTN and CD80/CD86 in adipocyte co-cultured TNBC cells. As a result, without affecting their expressions, knockdown of PCSK9 also reversed the contribution of adipocyte coculture to OPTN acetylation and OPTN-CD80/CD86 interactions in MDA-MB-231 cells (Fig. 6M). These results supported the importance of PCSK9 in influencing autophagic degradation of CD80/CD86 via the FAO-OPTN axis, thereby promoting immune evasion and tumor progression.

## Discussion

Cancer utilizes diverse mechanisms from the TME to support its growth. The TME in breast cancer is a complex and diverse ecosystem that includes the extracellular matrix, adipocytes, fibroblasts, and immune cells [8–14]. Adipocytes, particularly in individuals with obesity, are the most prevalent and possibly the most influential, yet the least well-studied elements within this TME [15, 18, 19]. However, some studies have demonstrated interactions between adipocytes and breast cancer cells [23], the reciprocal impacts between TNBC cells and surrounding adipocytes are largely unknown. In our study, we confirmed that TNBC cells actively induced lipolysis in adipocytes, effectively reprogramming them into CAAs. Interestingly, we observed that the transformation was furthered when TNBC cells were pre-educated by adipocytes. This observation also implies that in an obese environment, where adipocytes are more abundant and active, the interaction between tumor cells and adipocytes could be more pronounced.

Strong evidence suggests that the elevated inflammatory cytokines secreted by CAAs likely contribute to an enhanced inflammatory milieu within the TME, supporting various tumor progression [16, 18–20, 66–70]. Here, we also proved that adipocyte coculture contributed to TNBC cell proliferation and migration, and a high-fat diet facilitated TNBC tumor growth and metastasis as well. In tumor cells, co-culture resulted in direct lipid transfer from adipocytes to TNBC cells, leading to metabolic reprogramming, a hallmark of cancer [71]. In detail, we found that adipocyte-educated TNBC cells exhibited enhanced FAO, leading to elevated ATP production and acetyl-CoA levels. The transition from glycolysis to FAO illustrated how TNBC cells adjust their metabolism for energy production and antioxidant defense to sustain tumor cell rapid growth [72, 73].

We then aimed to explore the key mediators of crosstalk between adipocytes and TNBC cells. Previous studies have identified EVs as crucial players in cell-to-cell communication [74–79]. We demonstrated that exosomes derived from tumor cells effectively recapitulated the effects of co-culture with TNBC cells in converting adipocytes into CAAs. This indicated that TNBC cells release exosomes that efficiently convert adipocytes into CAAs. Fortunately, we identified a critical actor in lipid metabolism, PCSK9, as the molecular crux transferred via exosomes from TNBC cells to adipocytes. PCSK9 is a key regulator of cholesterol metabolism. Emerging evidence suggests that PCSK9 also plays an important role in the development and progression of various human cancers, including breast cancer [36, 80], hepatocellular carcinoma [37], colorectal cancer [38], and melanoma [39, 40]. This makes PCSK9 a potential target for cancer therapy [37, 41–45]. Mechanistically, studies have revealed that PCSK9 functions in cancer development via modulating signaling pathways such as PI3K/Akt, MAPK, and Wnt/β-catenin [38, 81, 82]. In the current study, we further expanded the biological roles of exosomal PCSK9 from TNBC cells in the transformation of adipocytes into CAAs, which, in turn, create a tumor-promoting microenvironment. In addition, we found that G3BP1 stabilized PCSK9 through its rG4 structure. Moreover, as suggested by previous findings [50, 51], we verified that adipocyte coculture-elevated FAO contributed to G3BP1 expression in TNBC cells by activating NF-κB. Together, adipocyte-induced FAO in TNBC cells further strengthened PCSK9 expression via reinforcing G3BP1-mediated stabilization, which explained the previously observed phenomena that adipocyte-educated TNBC cells have stronger induction for adipocyte-CAA transformation than parental TNBC cells. However, the detailed mechanisms by which PCSK9 induces CAAs remain to be investigated.

Presently, groundbreaking discoveries have established PCSK9’s multifaceted roles, including its control of pancreatic cancer metastatic organotropism [83], and mediation of germline-associated breast cancer metastasis [80]. Of interest, recent comprehensive studies have unraveled the intricacies of PCSK9’s involvement in tumor immunity and immunotherapy [54–59]. For example, depletion of PCSK9 attenuates tumor growth in syngeneic mice, and antibody-mediated neutralization of PCSK9 enhances anti-PD1 therapy, overcoming tumor resistance to anti-PD1 [54]. In our study, we found that tumors with increased PCSK9 expression had lower lymphocytic infiltrates compared to those in the control group. Mechanistic investigations revealed that adipocyte co-culture potentiated FAO to induce the autophagic degradation of CD80/CD86 through an OPTN-dependent manner in TNBC cells. Moreover, we found that adipocyte-reinforced FAO promoted OPTN acetylation in UBD and thereby facilitated its recognition by CD80/CD86 in TNBC cells. Such findings are consistent with those showing that acetylation of the UBD of autophagy receptors, such as p62 [64], can generally strengthen their affinity for ubiquitinated substrates [65]. Of course, we also discovered that PCSK9 positively regulates the interaction between OPTN and CD80/CD86 in TNBC cells, given its importance in inducing adipocyte-CAA transformation. Our findings suggested that PCSK9 may play a crucial role in tumor immunity by facilitating the degradation of CD80/CD86 through inducing CAA generation and CAA-elevated FAO in TNBC cells. Moreover, the clinical data further supported the suppression of PCSK9 on CD80/CD86 and its significance in inducing CAAs in TNBC, thus contributing to TNBC progression and immune evasion. The present work highlights that targeting PCSK9 might be a promising strategy to combat TNBC or enhance the efficacy of immune therapy.

## Conclusions

In summary, our study elucidates how PCSK9 mediates interactions between TNBC cells and adipocytes. TNBC cells induce the formation of CAAs through transferring exosomal PCSK9, thereby creating a tumor-promoting environment. In return, CAAs aggravate FAO in TNBC cells, thereby reciprocally strengthening PCSK9 expression by potentiating G3BP1-mediated stabilization and enhancing tumor immune evasion by facilitating OPTN-mediated autophagic degradation of CD80/CD86, ultimately accelerating tumor progression and metastasis (Fig. 6N). Further exploration of the PCSK9 pathway could revolutionize TNBC treatment and enable the optimization of therapeutic strategies.

## Supplementary information

Supplementary figure legends

Figure S1

Figure S2

Figure S3

Figure S4

Figure S5

Original Data 1

Original Data 2

Original Data 3

Original Data 4

Original Data 5

## Data Availability

Research data can be obtained from the corresponding author if necessary

## References

[1] Foulkes WD, Smith IE, Reis-Filho JS. Triple-negative breast cancer. N Engl J Med. 2010;363:1938–48. 10.1056/NEJMra1001389 21067385

[2] Collignon J, Lousberg L, Schroeder H, Jerusalem G. Triple-negative breast cancer: treatment challenges and solutions. Breast cancer (Dove Med Press). 2016;8:93–107. 10.2147/BCTT.S69488 PMC4881925 27284266

[3] Derakhshan F, Reis-Filho JS. Pathogenesis of triple-negative breast cancer. Annu Rev Pathol. 2022;17:181–204. 10.1146/annurev-pathol-042420-093238 PMC9231507 35073169

[4] Schmid P, Adams S, Rugo HS, Schneeweiss A, Barrios CH, Iwata H, et al. Atezolizumab and nab-paclitaxel in advanced triple-negative breast cancer. N Engl J Med. 2018;379:2108–21. 10.1056/NEJMoa1809615 30345906

[5] Cortes J, Cescon DW, Rugo HS, Nowecki Z, Im SA, Yusof MM, et al. Pembrolizumab plus chemotherapy versus placebo plus chemotherapy for previously untreated locally recurrent inoperable or metastatic triple-negative breast cancer (KEYNOTE-355): a randomised, placebo-controlled, double-blind, phase 3 clinical trial. Lancet (Lond, Engl). 2020;396:1817–28. 10.1016/S0140-6736(20)32531-9 33278935

[6] Schmid P, Cortes J, Pusztai L, McArthur H, Kümmel S, Bergh J, et al. Pembrolizumab for early triple-negative breast cancer. N Engl J Med. 2020;382:810–21. 10.1056/NEJMoa1910549 32101663

[7] Martin JD, Cabral H, Stylianopoulos T, Jain RK. Improving cancer immunotherapy using nanomedicines: progress, opportunities and challenges. Nat Rev Clin Oncol. 2020;17:251–66. 10.1038/s41571-019-0308-z PMC8272676 32034288

[8] Joyce JA, Pollard JW. Microenvironmental regulation of metastasis. Nat Rev Cancer. 2009;9:239–52. 10.1038/nrc2618 PMC3251309 19279573

[9] Quail DF, Joyce JA. Microenvironmental regulation of tumor progression and metastasis. Nat Med. 2013;19:1423–37. 10.1038/nm.3394 PMC3954707 24202395

[10] DeBerardinis RJ. Tumor microenvironment, metabolism, and immunotherapy. N Engl J Med. 2020;382:869–71. 10.1056/NEJMcibr1914890 32101671

[11] de Visser KE, Joyce JA. The evolving tumor microenvironment: From cancer initiation to metastatic outgrowth. Cancer Cell. 2023;41:374–403. 10.1016/j.ccell.2023.02.016 36917948

[12] Binnewies M, Roberts EW, Kersten K, Chan V, Fearon DF, Merad M, et al. Understanding the tumor immune microenvironment (TIME) for effective therapy. Nat Med. 2018;24:541–50. 10.1038/s41591-018-0014-x PMC5998822 29686425

[13] Danenberg E, Bardwell H, Zanotelli VRT, Provenzano E, Chin SF, Rueda OM, et al. Breast tumor microenvironment structures are associated with genomic features and clinical outcome. Nat Genet. 2022;54:660–9. 10.1038/s41588-022-01041-y PMC7612730 35437329

[14] Song Y, Na H, Lee SE, Kim YM, Moon J, Nam TW, et al. Dysfunctional adipocytes promote tumor progression through YAP/TAZ-dependent cancer-associated adipocyte transformation. Nat Commun. 2024;15:4052. 10.1038/s41467-024-48179-3 PMC11094189 38744820

[15] Wiseman BS, Werb Z. Stromal effects on mammary gland development and breast cancer. Science (N Y, NY). 2002;296:1046–9. 10.1126/science.1067431 PMC2788989 12004111

[16] Dirat B, Bochet L, Dabek M, Daviaud D, Dauvillier S, Majed B, et al. Cancer-associated adipocytes exhibit an activated phenotype and contribute to breast cancer invasion. Cancer Res. 2011;71:2455–65. 10.1158/0008-5472.CAN-10-3323 21459803

[17] Zhu Q, Zhu Y, Hepler C, Zhang Q, Park J, Gliniak C, et al. Adipocyte mesenchymal transition contributes to mammary tumor progression. Cell Rep. 2022;40:111362. 10.1016/j.celrep.2022.111362 PMC9533474 36103820

[18] Choi J, Cha YJ, Koo JS. Adipocyte biology in breast cancer: from silent bystander to active facilitator. Prog Lipid Res. 2018;69:11–20. 10.1016/j.plipres.2017.11.002 29175445

[19] Wu Q, Li B, Li Z, Li J, Sun S, Sun S. Cancer-associated adipocytes: key players in breast cancer progression. J Hematol Oncol. 2019;12:95. 10.1186/s13045-019-0778-6 PMC6734503 31500658

[20] Gao Y, Chen X, He Q, Gimple RC, Liao Y, Wang L, et al. Adipocytes promote breast tumorigenesis through TAZ-dependent secretion of Resistin. Proc Natl Acad Sci USA. 2020;117:33295–304. 10.1073/pnas.2005950117 PMC7776784 33318171

[21] Mukherjee A, Bezwada D, Greco F, Zandbergen M, Shen T, Chiang CY, et al. Adipocytes reprogram cancer cell metabolism by diverting glucose towards glycerol-3-phosphate thereby promoting metastasis. Nat Metab. 2023;5:1563–77. 10.1038/s42255-023-00879-8 PMC12175003 37653041

[22] Lengyel E, Makowski L, DiGiovanni J, Kolonin MG. Cancer as a matter of fat: the crosstalk between adipose tissue and tumors. Trends cancer. 2018;4:374–84. 10.1016/j.trecan.2018.03.004 PMC5932630 29709261

[23] Balaban S, Shearer RF, Lee LS, van Geldermalsen M, Schreuder M, Shtein HC, et al. Adipocyte lipolysis links obesity to breast cancer growth: adipocyte-derived fatty acids drive breast cancer cell proliferation and migration. Cancer Metab. 2017;5:1. 10.1186/s40170-016-0163-7 PMC5237166 28101337

[24] Gyamfi J, Yeo JH, Kwon D, Min BS, Cha YJ, Koo JS, et al. Interaction between CD36 and FABP4 modulates adipocyte-induced fatty acid import and metabolism in breast cancer. NPJ Breast Cancer. 2021;7:129. 10.1038/s41523-021-00324-7 PMC8463699 34561446

[25] Huang R, Wang Z, Hong J, Wu J, Huang O, He J, et al. Targeting cancer-associated adipocyte-derived CXCL8 inhibits triple-negative breast cancer progression and enhances the efficacy of anti-PD-1 immunotherapy. Cell Death Dis. 2023;14:703. 10.1038/s41419-023-06230-z PMC10613226 37898619

[26] Wu Q, Li B, Li J, Sun S, Yuan J, Sun S. Cancer-associated adipocytes as immunomodulators in cancer. Biomark Res. 2021;9:2. 10.1186/s40364-020-00257-6 PMC7792018 33413697

[27] Zhang C, Yue C, Herrmann A, Song J, Egelston C, Wang T, et al. STAT3 activation-induced fatty acid oxidation in CD8( + ) T effector cells is critical for obesity-promoted breast tumor growth. Cell Metab. 2020;31:148–61.e5. 10.1016/j.cmet.2019.10.013 PMC6949402 31761565

[28] Ewertz M, Jensen MB, Gunnarsdóttir K, Højris I, Jakobsen EH, Nielsen D, et al. Effect of obesity on prognosis after early-stage breast cancer. J Clin Oncol : J Am Soc Clin Oncol. 2011;29:25–31. 10.1200/JCO.2010.29.7614 21115856

[29] Kothari C, Diorio C, Durocher F. The importance of breast adipose tissue in breast cancer. Int J Mol Sci. 2020;21:5760. 10.3390/ijms21165760 PMC7460846 32796696

[30] Ringel AE, Drijvers JM, Baker GJ, Catozzi A, García-Cañaveras JC, Gassaway BM, et al. Obesity shapes metabolism in the tumor microenvironment to suppress anti-tumor immunity. Cell. 2020;183:1848–66.e26. 10.1016/j.cell.2020.11.009 PMC8064125 33301708

[31] Iyengar NM, Gucalp A, Dannenberg AJ, Hudis CA. Obesity and cancer mechanisms: tumor microenvironment and inflammation. J Clin Oncol : J Am Soc Clin Oncol. 2016;34:4270–6. 10.1200/JCO.2016.67.4283 PMC5562428 27903155

[32] Hauser S, Sommerfeld P, Wodtke J, Hauser C, Schlitterlau P, Pietzsch J, et al. Application of a fluorescence anisotropy-based assay to quantify transglutaminase 2 activity in cell lysates. Int J Mol Sci. 2022;23:4475. 10.3390/ijms23094475 PMC9104438 35562866

[33] Iacobucci G. Sixty seconds on … concussion in football. BMJ (Clin Res ed). 2021;373:n1119. 10.1136/bmj.n1119 33926890

[34] Wang HX, Gires O. Tumor-derived extracellular vesicles in breast cancer: from bench to bedside. Cancer Lett. 2019;460:54–64. 10.1016/j.canlet.2019.06.012 31233837

[35] Xu R, Rai A, Chen M, Suwakulsiri W, Greening DW, Simpson RJ. Extracellular vesicles in cancer - implications for future improvements in cancer care. Nat Rev Clin Oncol. 2018;15:617–38. 10.1038/s41571-018-0036-9 29795272

[36] Wong Chong E, Joncas FH, Seidah NG, Calon F, Diorio C, Gangloff A. Circulating levels of PCSK9, ANGPTL3 and Lp(a) in stage III breast cancers. BMC cancer. 2022;22:1049. 10.1186/s12885-022-10120-6 PMC9535963 36203122

[37] Sun Y, Zhang H, Meng J, Guo F, Ren D, Wu H, et al. S-palmitoylation of PCSK9 induces sorafenib resistance in liver cancer by activating the PI3K/AKT pathway. Cell Rep. 2022;40:111194. 10.1016/j.celrep.2022.111194 35977495

[38] Wong CC, Wu JL, Ji F, Kang W, Bian X, Chen H, et al. The cholesterol uptake regulator PCSK9 promotes and is a therapeutic target in APC/KRAS-mutant colorectal cancer. Nat Commun. 2022;13:3971. 10.1038/s41467-022-31663-z PMC9270407 35803966

[39] Sun X, Essalmani R, Day R, Khatib AM, Seidah NG, Prat A. Proprotein convertase subtilisin/kexin type 9 deficiency reduces melanoma metastasis in liver. Neoplasia (N Y, NY). 2012;14:1122–31. 10.1593/neo.121252 PMC3540939 23308045

[40] Suh JM, Son Y, Yoo JY, Goh Y, Seidah NG, Lee S, et al. Proprotein convertase subtilisin/kexin Type 9 is required for Ahnak-mediated metastasis of melanoma into lung epithelial cells. Neoplasia (N Y, NY). 2021;23:993–1001. 10.1016/j.neo.2021.07.007 PMC8350332 34352405

[41] Seidah NG, Prat A. The multifaceted biology of PCSK9. Endocr Rev. 2022;43:558–82. 10.1210/endrev/bnab035 PMC9113161 35552680

[42] Fang S, Yarmolinsky J, Gill D, Bull CJ, Perks CM, Davey Smith G, et al. Association between genetically proxied PCSK9 inhibition and prostate cancer risk: A Mendelian randomisation study. PLoS Med. 2023;20:e1003988. 10.1371/journal.pmed.1003988 PMC9810198 36595504

[43] Mahboobnia K, Pirro M, Marini E, Grignani F, Bezsonov EE, Jamialahmadi T, et al. PCSK9 and cancer: rethinking the link. Biomed Pharmacother. 2021;140:111758. 10.1016/j.biopha.2021.111758 34058443

[44] Oza PP, Kashfi K. The evolving landscape of PCSK9 inhibition in cancer. Eur J Pharm. 2023;949:175721. 10.1016/j.ejphar.2023.175721 PMC10229316 37059376

[45] Yang QC, Wang S, Liu YT, Song A, Wu ZZ, Wan SC, et al. Targeting PCSK9 reduces cancer cell stemness and enhances antitumor immunity in head and neck cancer. iScience. 2023;26:106916. 10.1016/j.isci.2023.106916 PMC10250824 37305703

[46] Dumas L, Herviou P, Dassi E, Cammas A, Millevoi S. G-quadruplexes in RNA biology: recent advances and future directions. Trends Biochem Sci. 2021;46:270–83. 10.1016/j.tibs.2020.11.001 33303320

[47] Cammas A, Millevoi S. RNA G-quadruplexes: emerging mechanisms in disease. Nucleic Acids Res. 2017;45:1584–95. 10.1093/nar/gkw1280 PMC5389700 28013268

[48] Kharel P, Ivanov P. RNA G-quadruplexes and stress: emerging mechanisms and functions. Trends Cell Biol. 2024;34:771–84. 10.1016/j.tcb.2024.01.005 PMC12069074 38341346

[49] He X, Yuan J, Wang Y. G3BP1 binds to guanine quadruplexes in mRNAs to modulate their stabilities. Nucleic Acids Res. 2021;49:11323–36. 10.1093/nar/gkab873 PMC8565330 34614161

[50] Jiang N, Xie B, Xiao W, Fan M, Xu S, Duan Y, et al. Fatty acid oxidation fuels glioblastoma radioresistance with CD47-mediated immune evasion. Nat Commun. 2022;13:1511. 10.1038/s41467-022-29137-3 PMC8938495 35314680

[51] Noma N, Simizu S, Kambayashi Y, Kabe Y, Suematsu M, Umezawa K. Involvement of NF-κB-mediated expression of galectin-3-binding protein in TNF-α-induced breast cancer cell adhesion. Oncol Rep. 2012;27:2080–4. 10.3892/or.2012.1733 22447108

[52] Castello A, Fischer B, Frese CK, Horos R, Alleaume AM, Foehr S, et al. Comprehensive identification of RNA-binding domains in human cells. Mol cell. 2016;63:696–710. 10.1016/j.molcel.2016.06.029 PMC5003815 27453046

[53] Bouche C, Quail DF. Fueling the tumor microenvironment with cancer-associated adipocytes. Cancer Res. 2023;83:1170–2. 10.1158/0008-5472.CAN-23-0505 37057599

[54] Liu X, Bao X, Hu M, Chang H, Jiao M, Cheng J, et al. Inhibition of PCSK9 potentiates immune checkpoint therapy for cancer. Nature. 2020;588:693–8. 10.1038/s41586-020-2911-7 PMC7770056 33177715

[55] Gu Y, Lin X, Dong Y, Wood G, Seidah NG, Werstuck G, et al. PCSK9 facilitates melanoma pathogenesis via a network regulating tumor immunity. J Exp Clin cancer Res : CR. 2023;42:2. 10.1186/s13046-022-02584-y PMC9806914 36588164

[56] Gao X, Yi L, Jiang C, Li S, Wang X, Yang B, et al. PCSK9 regulates the efficacy of immune checkpoint therapy in lung cancer. Front Immunol. 2023;14:1142428. 10.3389/fimmu.2023.1142428 PMC10070680 37025995

[57] Wang H, Zhang X, Zhang Y, Shi T, Zhang Y, Song X, et al. Targeting PCSK9 to upregulate MHC-II on the surface of tumor cells in tumor immunotherapy. BMC cancer. 2024;24:445. 10.1186/s12885-024-12148-2 PMC11007992 38600469

[58] Wang R, Liu H, He P, An D, Guo X, Zhang X, et al. Inhibition of PCSK9 enhances the antitumor effect of PD-1 inhibitor in colorectal cancer by promoting the infiltration of CD8( + ) T cells and the exclusion of Treg cells. Front Immunol. 2022;13:947756. 10.3389/fimmu.2022.947756 PMC9393481 36003387

[59] Crunkhorn S. Blocking PCSK9 enhances immune checkpoint therapy. Nat Rev Drug Discov. 2021;20:20. 10.1038/d41573-020-00208-8 33262511

[60] Bugeon L, Dallman MJ. Costimulation of T cells. Am J Respir Crit Care Med. 2000;162:S164–8. 10.1164/ajrccm.162.supplement_3.15tac5 11029388

[61] Rahighi S, Ikeda F, Kawasaki M, Akutsu M, Suzuki N, Kato R, et al. Specific recognition of linear ubiquitin chains by NEMO is important for NF-kappaB activation. Cell. 2009;136:1098–109. 10.1016/j.cell.2009.03.007 19303852

[62] Li F, Xu D, Wang Y, Zhou Z, Liu J, Hu S, et al. Structural insights into the ubiquitin recognition by OPTN (optineurin) and its regulation by TBK1-mediated phosphorylation. Autophagy. 2018;14:66–79. 10.1080/15548627.2017.1391970 PMC5846504 29394115

[63] Li F, Xie X, Wang Y, Liu J, Cheng X, Guo Y, et al. Structural insights into the interaction and disease mechanism of neurodegenerative disease-associated optineurin and TBK1 proteins. Nat Commun. 2016;7:12708. 10.1038/ncomms12708 PMC5027247 27620379

[64] You Z, Jiang WX, Qin LY, Gong Z, Wan W, Li J, et al. Requirement for p62 acetylation in the aggregation of ubiquitylated proteins under nutrient stress. Nat Commun. 2019;10:5792. 10.1038/s41467-019-13718-w PMC6923396 31857589

[65] Xu Y, Wan W. Acetylation in the regulation of autophagy. Autophagy. 2023;19:379–87. 10.1080/15548627.2022.2062112 PMC9851266 35435793

[66] Nickel A, Blücher C, Kadri OA, Schwagarus N, Müller S, Schaab M, et al. Adipocytes induce distinct gene expression profiles in mammary tumor cells and enhance inflammatory signaling in invasive breast cancer cells. Sci Rep. 2018;8:9482. 10.1038/s41598-018-27210-w PMC6013441 29930291

[67] Spaziante G. [The problem of noises in hospitals. I. Phonometric data in various environments and in various conditions]. Riv Ital d’Ig. 1966;26:468–511. 5995871

[68] Zhao H, Wu L, Yan G, Chen Y, Zhou M, Wu Y, et al. Inflammation and tumor progression: signaling pathways and targeted intervention. Signal Transduct Target Ther. 2021;6:263. 10.1038/s41392-021-00658-5 PMC8273155 34248142

[69] Coussens LM, Werb Z. Inflammation and cancer. Nature. 2002;420:860–7. 10.1038/nature01322 PMC2803035 12490959

[70] Deng T, Lyon CJ, Bergin S, Caligiuri MA, Hsueh WA. Obesity, Inflammation, and Cancer. Annu Rev Pathol. 2016;11:421–49. 10.1146/annurev-pathol-012615-044359 27193454

[71] Hanahan D. Hallmarks of cancer: new dimensions. Cancer Discov. 2022;12:31–46. 10.1158/2159-8290.CD-21-1059 35022204

[72] Röhrig F, Schulze A. The multifaceted roles of fatty acid synthesis in cancer. Nat Rev Cancer. 2016;16:732–49. 10.1038/nrc.2016.89 27658529

[73] Martin-Perez M, Urdiroz-Urricelqui U, Bigas C, Benitah SA. The role of lipids in cancer progression and metastasis. Cell Metab. 2022;34:1675–99. 10.1016/j.cmet.2022.09.023 36261043

[74] Moraes JA, Encarnação C, Franco VA, Xavier Botelho LG, Rodrigues GP, Ramos-Andrade I, et al. Adipose tissue-derived extracellular vesicles and the tumor microenvironment: revisiting the hallmarks of cancer. Cancers. 2021;13:3328. 10.3390/cancers13133328 PMC8268128 34283044

[75] Zhang C, Qin C, Dewanjee S, Bhattacharya H, Chakraborty P, Jha NK, et al. Tumor-derived small extracellular vesicles in cancer invasion and metastasis: molecular mechanisms, and clinical significance. Mol Cancer. 2024;23:18. 10.1186/s12943-024-01932-0 PMC10797874 38243280

[76] Clancy JW, D’Souza-Schorey C. Tumor-derived extracellular vesicles: multifunctional entities in the tumor microenvironment. Annu Rev Pathol. 2023;18:205–29. 10.1146/annurev-pathmechdis-031521-022116 PMC10410237 36202098

[77] Kenific CM, Wang G, Lyden D. Tumor extracellular vesicles impede interferon alert responses. Cancer Cell. 2019;35:3–5. 10.1016/j.ccell.2018.12.006 30645974

[78] Kalluri R, McAndrews KM. The role of extracellular vesicles in cancer. Cell. 2023;186:1610–26. 10.1016/j.cell.2023.03.010 PMC10484374 37059067

[79] Becker A, Thakur BK, Weiss JM, Kim HS, Peinado H, Lyden D. Extracellular vesicles in cancer: cell-to-cell mediators of metastasis. Cancer Cell. 2016;30:836–48. 10.1016/j.ccell.2016.10.009 PMC5157696 27960084

[80] Mei W, Faraj Tabrizi S, Godina C, Lovisa AF, Isaksson K, Jernström H, et al. A commonly inherited human PCSK9 germline variant drives breast cancer metastasis via LRP1 receptor. Cell. 2025;188:371–89.e28. 10.1016/j.cell.2024.11.009 PMC11770377 39657676

[81] Wang L, Li S, Luo H, Lu Q, Yu S. PCSK9 promotes the progression and metastasis of colon cancer cells through regulation of EMT and PI3K/AKT signaling in tumor cells and phenotypic polarization of macrophages. J Exp Clin cancer Res : CR. 2022;41:303. 10.1186/s13046-022-02477-0 PMC9563506 36242053

[82] Xu B, Li S, Fang Y, Zou Y, Song D, Zhang S, et al. Proprotein convertase subtilisin/kexin type 9 promotes gastric cancer metastasis and suppresses apoptosis by facilitating MAPK signaling pathway through HSP70 up-regulation. Front Oncol. 2020;10:609663. 10.3389/fonc.2020.609663 PMC7817950 33489919

[83] Rademaker G, Hernandez GA, Seo Y, Dahal S, Miller-Phillips L, Li AL, et al. PCSK9 drives sterol-dependent metastatic organ choice in pancreatic cancer. Nature. 2025;643:1381–90. 10.1038/s41586-025-09017-8 PMC13064930 40399683

