# Supplementary material for: Tumor-derived PCSK9-enriched exosomes reprogram adipocytes to drive metabolic dysregulation and immune evasion in triple-negative breast cancer
Source: Cell Death Dis. 2026 Jul 3;17(1):827. doi: 10.1038/s41419-026-09027-y (PMC13601526; doi:10.1038/s41419-026-09027-y)
Supplement: Supplementary file 1 [file 41419_2026_9027_MOESM1_ESM.docx]

**Figure S1. The correlations of CAA markers with TNBC patients’ prognoses.**

**A-B.** Kaplan-Meier plotter indicated the overall survival of TNBC patients with high or low IL-6 (A) or MMP11 (B) expression.

**Figure S2. TNBC Exosomes Promote Tumor-Associated Adipocyte Formation via PCSK9**

**A.** TEM analysis of exosomes from TNBC cells. **B.** NTA analysis of exosome diameter from TNBC cells. **C.** Western blot analysis of exosome markers. **D-E.** RT-qPCR and western blot analyses of PCSK9 expression changes in MDA-MB-231 cells under different transfections. **F.** Western blot analysis of PCSK9 expression in exosomes derived from tumor cells with PCSK9 knockdown or re-expression. *Statistical significance: ^**^P < 0.01*

**Figure S3. High-Fat Diet Promotes Aggressive Phenotypes in Triple-Negative Breast Cancer**

**A.** Fat weight measurement from different diet groups. **B.** Blood glucose level measurement from different diet groups. **C.** FAO levels from different diet groups. **D.** IHC was used to detect the expression of Ki67 in tumor tissues from the standard diet group and the high-fat diet group. Gross morphology of tumors from different diet groups.  *Statistical significance: ^*^P < 0.05, ^**^P < 0.01*

**Figure S4. G3BP1 Binds to the rG4 Structure of PCSK9**

**A.** Sequences of PCSK9 mRNA. **B.** Western blot detected the changes in acetylated RelA (RelA-ac) and total RelA in the indicated MDA-MB-231 cells. **C.** The activity of the NF-κB pathway was tested using a Luciferase reporter assay. **D.** The level of G3BP1 in MDA-MB-231 cells with adipocyte co-culture or NF-κB inhibitor treatment was estimated via western blot. **E.** Co-IP analysis of rG4 binding sites. **F.** RT-qPCR and Western blot verification of G3BP1 interference efficiency. **G.** Western blot analysis of the effect of G3BP1 knockout on PCSK9 expression. *Statistical significance: ^**^P < 0.01.*

**Figure S5. PCSK9 is associated with T cell infiltration in basal-like tumors**

The Timer diagram shows that PCSK9 is associated with T cell infiltration in basal-like tumors*.*
